# Adaptation to DNA Damage, an Asymptotic Approach for a Cooperative Non-local System

**DOI:** 10.1007/s10440-022-00501-1

**Published:** 2022-06-21

**Authors:** Alexis Léculier, Pierre Roux

**Affiliations:** 1 Laboratoire Jacques-Louis Lions (LJLL), Sorbonne Université, 75205 Paris Cedex 06, France; 2 Mathematical Institute, University of Oxford, OX2 6GG Oxford, UK

**Keywords:** Adaptive evolution, Cooperative system, Lotka-Volterra equation, Hamilton-Jacobi equation, Viscosity solutions, 35K60, 82C31, 92B20, 35Q84

## Abstract

Following previous works about integro-differential equations of parabolic type modelling the Darwinian evolution of a population, we study a two-population system in the cooperative case. First, we provide a theoretical study of the limit of rare mutations and we prove that the limit is described by a constrained Hamilton-Jacobi equation. This equation is given by an eigenvalue of a matrix which accounts for the diffusion parameters and the coefficients of the system. Then, we focus on a particular application: the understanding of a phenomenon called Adaptation to DNA damage. In this framework, we provide several numerical simulations to illustrate our theoretical results and investigate mathematical and biological questions.

## Introduction

A common way to investigate evolutionary dynamics [11, 22] is to model populations structured by a phenotypical trait with non-local partial differential equations [5, 6, 8]. This methodology has the advantage of studying not only the final situation but also the fitness landscape and the possible evolutionary paths in a given setting [42]. In those kind of models, the organisms are described by a trait $x\in \mathbb{R}^{n}$ and their density $n(x,t)$ expands or decays in function of both $x$ and the competition with other individuals. A simple possibility to represent mutations along the trait $x$ is to use a Laplacian:

1.1 $$ \dfrac{\partial n}{\partial t}(x,t) = \Delta n(x,t) + n(x,t)R(x,N(t)), \qquad N(t) = \int _{\mathbb{R}^{n}} n(x,t) dx. $$

This type of model can be derived from individual based stochastic models in the large population limit [15, 16].

In many works (*e.g.* [5, 6, 8, 10]), the authors introduce a parameter $\varepsilon >0$ which provides a way to study the asymptotic limit of the model in the regime of small mutations and long time [12]. This procedure relies upon a Hamilton-Jacobi approach and was extensively investigated for system (1.2). It consists in making the change of variable

$$ (x, t) = \left ( \frac{x}{\varepsilon} ,\frac{t}{ \varepsilon } \right ), $$

which leads to the equation

1.2 $$ \varepsilon \dfrac{\partial n^{\varepsilon}}{\partial t}(x,t) = \varepsilon ^{2}\Delta n^{\varepsilon}(x,t) + n^{\varepsilon}(x,t)R(x,N_{\varepsilon}(t)), \qquad N_{\varepsilon}(t) = \int _{\mathbb{R}^{n}} n^{\varepsilon}(x,t) dx. $$

This change of variables allows to catch the effective behaviour of the solutions in large timescales. It can be understood heuristically as follows. The parameter $\varepsilon $ in front of the time derivative accelerates the time meanwhile the parameter $\varepsilon ^{2}$ in front of the mutation operator (the Laplacian here) avoids that individuals adapt to their environment too fast. Therefore, only the individuals with a trait “well-adapted” to the environment survive. This is why, we expect the solution to concentrate to a sum of Dirac mass centred at these well adapted traits. In a suitable mathematical setting, when $\varepsilon \to 0$, the solutions $n^{\varepsilon}$ of (1.2) concentrate into a sum of Dirac masses moving in time, and in the limit the location of emergent traits is driven by an Hamilton-Jacobi equation of the form

1.3 $$ \dfrac{\partial u}{\partial t} = |\nabla u |^{2} + R(x,N(t)), \qquad \max _{x\in \mathbb{R}^{n}} u(x,t) = 0. $$

This type of non-local model was intensively studied and applied to many different biological contexts, for example adaptation of cancer to treatment [17, 33, 34, 43], epigenetics changes [32], non-inherited antibiotic resistance [9] or more generally long-time evolutionary dynamics [24, 26, 37]. Finally, we underline that a more realistic approach is to use an integral term and a mutation kernel (see for instance [5, 39] and the references therein) since, in our case, it is tantamount to saying that the mutations are independent of birth.

### Adaptation to DNA Damage as a Modelling Drive for This Study

The theoretical study in this article is mainly motivated by a specific modelling challenge which is still poorly understood from an evolutionary dynamics point of view: the so-called “adaptation to DNA damage” phenomenon.^1^

When eukaryotic cells face damage to their DNA, specialised mechanisms come into play. The DNA damage checkpoint signalling pathway leads to stopping the cell cycle at the G2/M phase. Then, appropriate repair pathways are activated. These mechanisms are called the DNA damage response. However, the attempts of the cell at restoring its DNA integrity can fail: for example, the damage may be impossible to repair, the appropriate repair pathway may not be available at this stage of the cell cycle or the source of the damage might still be present, causing damage faster than the cell can repair.

In case repair fails for too long, cells will override the DNA damage checkpoint and resume cell division even though the damage is still present [30, 50]. This prevents the cells from being blocked in the cell cycle until they die. This phenomenon is called adaptation to DNA damage. Note that this is a metabolic adaptation occurring in each individual cell which is to be distinguished from the genetic adaptation of the whole cell population on longer timescales (see footnote 1).

Due to improper chromosome segregation [27], adapted cells have chromosomal instability and a high mortality rate, making adaptation a last resort mechanism after all repair options have already failed. The descendants of adapted cells can carry the damage and share the mortality rate of their parents, but adaptation also allows cells to have access to other repair pathways at later stages of the cell cycle; it also happens that one of the daughter cells of an adapted cell doesn’t suffer from the damage due to asymmetric segregation of the chromosomes [48, 51]. Both the success of repair and, if repair is unsuccessful, the survival after an adaptation event, depend on the type and location of the damage. Double-strand breaks are especially dangerous to the cell survival and have been used by experimental biologists to investigate repair mechanisms.

This leads to a hierarchy of cell fate decisions: repair is attempted first and then the cells adapt. In the budding yeast model organism [45], the cells will adapt at a variable timing, ranging from 5 to 15 hours, which is consistent with the fact that one of the slowest repair pathways, break-induced replication,^2^ takes around 5 hours to be fully attempted [35]. Common experiments to investigate adaptation rely upon mutant populations which cannot repair heat-induced or irradiation-induced damage, making adaptation and its specific timescales easier to characterise [25, 28]. These experiments suggest that adaptation is detrimental to the population and mutants who cannot adapt have better survival when the experimental conditions are made favorable again.

Given how dangerous adaptation to DNA damage can be for cells and their progeny, an important question is to understand what shapes the characteristics of the phenomenon. The two main components of the adaptation process are when it happens on average after the damage which we refer to as the timing of adaptation and how variable it is in time, which we call heterogeneity of adaptation. At the moment, biologists have very few ways of investigating experimentally how these features are selected by natural selection or constrained by chemical limitations. Most of the experiments are performed in controlled environment and little is known about what happens in the wild or during long timescales. Some reviews by experts in the field proposed that adaptation timing and heterogeneity could have been selected by evolution because they are optimal for long term survival of the populations in adverse and unpredictable conditions (*e.g.* [20]).

A first evolutionary model was proposed recently for adaptation to DNA damage in [44]. The authors derive formally from a stochastic compartment model an ODE model representing how a population of damaged cells recovers and fills the medium up to carrying capacity.

If we call $x$ the adaptation timing and $p$ the heterogeneity of adaptation, the authors of [44] assume that cells damaged at time 0 repair with a given rate $\alpha (t)$ and adapt with a rate $\beta (x,p,t)$. The unit of $x$ is in cell-cycle, which is about 1.5–2 hours depending on the environmental conditions. The parameter $p$ ranges from 0 (totally random) and $+\infty $ (totally deterministic). Based on experimental insights, they choose a logistic model for the adaptation rate after a time $t$ since the damage:

1.4 $$ \beta ( x, p , t) = \dfrac{\beta _{m}}{1+ \mathrm{e}^{-p(t- x )}}, $$

with $\beta _{m}>0$ the maximal value of the adaptation rate.

Using first the stochastic compartment model and then the deterministic ODE model, the authors prove that there is an optimal value for $x$ that allows for the fastest recovery of a fully damaged population. However, their optimisation procedure indicates that the optimal value for $p$ is $+\infty $, which is in contrast with what the experiments indicate: adaptation ranges between 5 and 15 hours. The authors then improve their model, taking into account the fact that if the source of damage is still present for some hours (heat, X-rays, chemicals in the medium,…) then the cells cannot repair at all because their repair capacity is overloaded by the continuous source of damage. For each run of their model, they draw a random variable determining when repair becomes possible after the damage. With this component and optimising for expectancy of survival, they find an optimal value for the heterogeneity parameter $p$.

As explained in [44], the selection of an optimal value for the heterogeneity parameter when the environment becomes unpredictable can be related to the more general concept of bet-hedging. Bet-hedging occurs when, on the long term, an isogenic population of organisms selects a phenotype that is suboptimal in any given environment but is optimal for maximising survival in average in an unpredictable environment [47, 49]. A classical example is reservoirs of ungerminated seeds in the soil: in favourable conditions it is optimal for all seeds to germinate as soon as possible, but seed banks allows the population to escape extinction in case of severe drought because ungerminated seeds are less affected [19].

A major drawback in the work of [44] is that their model considers only an isogenic population with fixed parameters $x$ and $p$, and then compares, depending on the parameter, the fitness of the population. Their work doesn’t model how the cells change their genetic traits nor how cells with different genetic profiles compete with each other in a same environment. Their model is also very rigid in terms of evolutionary timescales and assumes an initial damage to the whole population instead of continuous damage in time. The later is more realistic since DNA damage can also come from endogenous stochastic causes.

In this work, we propose a more general approach using non-local partial differential equations of the type of (1.1) and a rescaling procedure akin to the one used in (1.2). In Sect. 5, we explain in more details the model of [44] and we build upon it a more complex model consisting in three coupled non-local PDEs representing healthy, damaged and adapted cells: system (5.5)–(5.6)–(5.7)–(5.8) in Sect. 5.

This system being difficult to tackle both theoretically and numerically, we make the simplifying assumption of a quasi-stationary equation for damaged cells, which allows us to remove them from the system by assuming they are treated instantly and redirected to the healthy cells population, the adapted cells population or killed. We thereby obtain a simpler two-populations model describing the dynamics and genetic diffusion of healthy and adapted cells: system (5.10)–(5.11) in Sect. 5. In the theoretical part of this article, we provide a mathematical study of a more general class of two-populations non-local models that includes system (5.10)–(5.11). The next subsection describes this mathematical framework.

### A Model for Two Cooperative Populations Structured by a Phenotypical Trait

We propose to study through a Hamilton-Jacobi procedure a system of non-local PDEs modelling two cooperative populations structured by a same phenotypical trait $x\in \mathbb{R}_{+}$ and described by their densities $n_{1}^{\varepsilon}(x,t)$ and $n_{2}^{\varepsilon}(x,t)$. This model generalises the system (5.10)–(5.11). For this reason, we first perform the abstract mathematical study in the general case in order to have more tool for the subsequent modelling and numerical parts.

To the best of our knowledge, there is little research about the asymptotic behaviour of several specie non-local PDEs in evolutionary dynamics. Existing works in this direction focus, for instance, on the influence of a spatial domain [10], on organisms which specialise in order to consume particular resources [23], on a model for juvenile-adult population undergoing small mutations [14], on elliptic systems [29, 37, 41] for two species or on influence of a spatial domain [10].

The model we focus on writes

1.5 $$ \left \lbrace \begin{aligned} &\varepsilon \partial _{t} n_{1}^{\varepsilon}- \varepsilon ^{2} d_{1} \partial _{xx} n_{1}^{\varepsilon}= n_{1}^{\varepsilon}(r_{1}(x) - N_{\varepsilon}(t)) + \delta _{1}(x) n_{2}^{\varepsilon}\quad \text{ for } (x, t) \in \mathbb{R}^{+} \times \mathbb{R}^{+}, \\ &\varepsilon \partial _{t} n_{2}^{\varepsilon}- \varepsilon ^{2} d_{2} \partial _{xx} n_{2}^{\varepsilon}= n_{2}^{\varepsilon}(r_{2}(x) - N_{\varepsilon}(t) )+ \delta _{2}(x) n_{1}^{\varepsilon}\quad \text{ for } (x, t) \in \mathbb{R}^{+} \times \mathbb{R}^{+}, \\ &n_{1}(x, t=0) = n_{1,0}^{\varepsilon}(x), \qquad n_{2}^{\varepsilon}(x, t=0) = n_{2, 0}^{0}(x), \\ &\partial _{x} n_{1}(x=0, t) = 0, \qquad \partial _{x} n_{2}^{\varepsilon}(x=0, t) = 0, \\ &N_{\varepsilon}(t) = \int _{0}^{+\infty} \big( n_{1}^{\varepsilon}(x,t) + n_{2}^{\varepsilon}(x,t) \big)dx, \end{aligned} \right . $$

where $r_{1}(x)\geqslant 0$ and $r_{2}(x)\geqslant 0$ represent the intrinsic fitness of organisms with trait $x$ in the two populations. The terms $\delta _{1}(x)\geqslant 0$ and $\delta _{2}(x)\geqslant 0$ are cooperative terms (or, in our application in Sect. 5, conversion terms from one cell type to the other) between the two populations. The total number of cells $N_{\varepsilon}(t)$ represents the competition for resources. Since the system is cooperative (i.e. an increase in one of the two populations imply an increase in the other population), we expect that both population converge to a Dirac mass with a same trait as $\varepsilon \to 0$, even in the case where one population has a smaller mutation rate $d_{i}$ (notice that we allow in the forthcoming assumption (H1) $d_{i} = 0$ but $\max (d_{i} , d_{j}) >0$). Heuristically, the dynamics of the main phenotype of this population will be driven by the mutation of the other population and the exchange term.

The system can be summarised in the following compact form

1.6 $$ \varepsilon \partial _{t} \textbf{n}^{\varepsilon}- \varepsilon ^{2} \textbf{D} \partial _{xx} \textbf{n}^{\varepsilon}= \textbf{R}(x, N_{\varepsilon}) \textbf{n}^{\varepsilon}, $$

with Neuman boundary conditions: $\partial _{x} \textbf{n}^{\varepsilon}(x = 0, t) = \mathbf{0}$. Here $\textbf{n}^{\varepsilon}$ stands for the vector $(n_{1}^{\varepsilon}, n_{2}^{\varepsilon})^{T}$ and $\textbf{D}, \ \textbf{R}$ for the following operators:

1.7 $$ \textbf{D} = \begin{pmatrix} d_{1} & 0 \\ 0 & d_{2} \end{pmatrix} \quad \text{ and } \quad \textbf{R}(x, N) = \begin{pmatrix} r_{1}(x) - N & \delta _{1}(x) \\ \delta _{2}(x) & r_{2}(x) - N \end{pmatrix} . $$

First, we assume that

H1 $$ d_{1} , \ d_{2} \geq 0 \quad \text{ and } \quad (d_{1} , d_{2} ) \neq (0, 0). $$

Note that (H1) allows one of the two coefficients $d_{1},d_{2}$ being equal to 0, but not both at the same time. We will also assume that there exists $C_{R}, C_{\delta}>0$ such that

H2 $$ \begin{aligned} &\delta _{i}, r_{i} \in W^{2, \infty} \quad \text{ with } \quad \| r_{i} \|_{W^{2, \infty}} \leq C_{R} \quad \text{ and } \| \delta _{i} \|_{W^{2, \infty}} \leq C_{\delta}, \\ &\delta _{i} > 0, \quad \text{ and } \quad e^{C_{\delta}x}\delta _{i}(x) \underset{x \to +\infty}{\longrightarrow} +\infty . \end{aligned} $$

An other hypothesis is

H3 $$ \begin{aligned} \exists c_{N}, C_{N}>0: \qquad \forall x \in \mathbb{R}^{+}, \ & \min (r_{1}(x) + \delta _{2}(x) -c_{N}, \ r_{2}(x) +\delta _{1}(x) -c_{N}) \geq 0, \\ \forall x \in \mathbb{R}^{+}, \ &\max (r_{1}(x) + \delta _{2}(x) -C_{N}, \ r_{2}(x) + \delta _{1}(x) -C_{N}) \leq 0. \end{aligned} $$

Finally, we assume that both initial conditions satisfy:

H4 $$ \begin{aligned} &ce^{\frac{{-ax^{2}-c}}{\varepsilon}} \leq n_{i, 0}^{\varepsilon}(x) \leq Ce^{\frac{{-Ax + C}}{\varepsilon}} \qquad \text{ with } \quad a, A, c, C>0, \\ &c_{N} \leq N_{\varepsilon}(t=0) \leq C_{N} \quad \text{ and } \quad n_{i,0}^{\varepsilon}\text{ are uniformly Lipshitz}. \end{aligned} $$

#### Theorem 1.1

*Under the assumptions* (H1), (H2), (H3) *and* (H4), *there exists a solution* $\textbf{n}^{\varepsilon}$ *to* (1.5). *Moreover*, *we have*

$$ c_{N} \leq N_{\varepsilon}(t) \leq C_{N}. $$

The proof is an adaptation of the one presented in Appendix A of [8]. We provide it in the Appendix for the sake of completeness.

### The Main Mathematical Result

We adopt the classical approach for Hamilton-Jacobi equations: we perform the so-called Hopf-Cole transformation by defining

1.8 $$ u_{i}^{\varepsilon}= \varepsilon \ln (n_{i}^{\varepsilon}). $$

This approach is well adapted since we expect $n_{i}^{\varepsilon}$ to converge to a Dirac mass in the sens of the measure as $\varepsilon $ vanishes. In this order, it is sufficient to prove that $u_{i}^{\varepsilon}$ converges in some set of continuous functions $u_{i}$ with $u_{i}(x_{0},t_{0})=0$ for some $(x_{0}, t_{0})$ whereas $u_{i}(x,t)<0$ for $(x,t) \in B_{r}(x_{0}, t_{0}) \backslash \left \lbrace (x_{0}, t_{0}) \right \rbrace $. Performing the inverse transformation of the Hopf-Cole transform provides the desired result. Therefore, we rewrite (1.5) in the following form

1.9 $$ \left \lbrace \begin{aligned} & \partial _{t} u_{1}^{\varepsilon}- \varepsilon d_{1}\partial _{xx} u_{1}^{\varepsilon}- d_{1}[\partial _{x} u_{1}^{\varepsilon}]^{2}= (r_{1}(x) - N_{\varepsilon}(t)) + \delta _{1}(x) e^{ \frac{u_{2}^{\varepsilon}- u_{1}^{\varepsilon}}{\varepsilon}} \quad \text{ for } (x, t) \in \mathbb{R}^{+} \times \mathbb{R}^{+}, \\ &\partial _{t} u_{2}^{\varepsilon}- \varepsilon d_{2}\partial _{xx} u_{2}^{\varepsilon}- d_{2} [\partial _{x} u_{2}^{\varepsilon}]^{2}= (r_{2}(x) - N_{\varepsilon}(t)) + \delta _{2}(x) e^{ \frac{u_{1}^{\varepsilon}- u_{2}^{\varepsilon}}{\varepsilon}}\quad \text{ for } (x, t) \in \mathbb{R}^{+} \times \mathbb{R}^{+}, \\ &u_{1}^{\varepsilon}(x, t=0) = u_{1,0}(x), \qquad u_{2}^{\varepsilon}(x, t=0) = u_{2,0}(x), \\ &\partial _{x} u_{1}(x=0, t) = 0, \qquad \partial _{x} u_{2}^{\varepsilon}(x=0, t) = 0, \\ &N_{\varepsilon}(t) = \int _{0}^{+\infty} \left ( e^{ \frac{u_{1}^{\varepsilon}(x,t)}{\varepsilon}} + e^{ \frac{u_{2}^{\varepsilon}(x,t)}{\varepsilon}}\right ) dx. \end{aligned} \right . $$

Finally, following [7], we introduce the effective Hamiltonian as known as one of the eigenvalue of $\rho ^{2}\textbf{D} + \textbf{R}$ (associated to a constant sign eigen-vector):

1.10 $$ \mathcal{H}_{D}(\rho , N) =\frac{d_{1} + d_{2}}{2}\rho ^{2} + \frac{ r_{1} + r_{2} + \sqrt{[(d_{1} - d_{2})\rho ^{2} + (r_{1} - r_{2})]^{2} + 4 \delta _{1} \delta _{2} }}{2 } - N(t). $$

We introduce the *Hamiltonian fitness*

1.11 $$ r_{H}^{D} (x, \rho ) = \frac{ r_{1} + r_{2} + \sqrt{[(d_{1} - d_{2})\rho ^{2} + (r_{1} - r_{2})]^{2} + 4 \delta _{1} \delta _{2} }}{2 } $$

such that

$$ \mathcal{H}_{D}(\rho , N) =\frac{d_{1} + d_{2}}{2}\rho ^{2} + r_{H}^{D} (x,\rho ) - N. $$

We will denote $\psi ^{\rho}$ the corresponding principal eigen-vector:

1.12 $$ \psi ^{\rho}(x) = \begin{pmatrix} 1 \\ \frac{ (d_{1} - d_{2}) \rho ^{2} + (r_{1}(x) - r_{2}(x)) + \sqrt{((d_{1} - d_{2}) \rho ^{2} + r_{1}(x) - r_{2}(x))^{2} + 4 \delta _{1}(x) \delta _{2}(x) }}{2\delta _{2}(x)}\end{pmatrix} . $$

All the components of $\psi ^{\rho}$ can be chosen strictly positive. The other eigenvector, associated to the eigenvalue $\frac{ (d_{1} + d_{2})\rho ^{2} + r_{1} + r_{2} - \sqrt{ (d_{1} - d_{2})\rho ^{2} + [(r_{1} - r_{2}) ]^{2} + 4 \delta _{1} \delta _{2} }}{2 } - N(t)$, has a positive and a negative component.

#### Theorem 1.2

*Under the hypotheses* (H1), (H2), (H3) *and* (H4), *there hold*

1. *The sequence* $(N_{\varepsilon})_{\varepsilon > 0}$ *converges to a non*-*decreasing function* $N \in L^{\infty}(\,]0, +\infty [\,)$ *as* $\varepsilon \to 0$ *with*
  $$ c_{N} \leq N(t) \leq C_{N}.$$
2. *The sequence* $(u_{i}^{\varepsilon})_{\varepsilon >0, \ i \in \left \lbrace 1,2 \right \rbrace}$ *converges locally uniformly to a same continuous function* $u$, *with* $u$ *a viscosity solution of*
  1.13 $$ \left \lbrace \begin{aligned} &\partial _{t} u = \mathcal{H}_{D}(\partial _{x} u , N) && \textit{ for } (x,t) \in \mathbb{R}_{+} \times \, ]0, +\infty [, \\ &- \partial _{x} u (x=0, t) = 0 && \textit{ for } t>0, \\ &\underset{ x \in \mathbb{R}_{+}}{\max} \ u(x,t) = 0 , \\ &u(x,t=0) = \underset{\varepsilon \to 0}{\lim} \ u_{i}^{\varepsilon}(x, t=\varepsilon ). \end{aligned} \right . $$
3. *The sequence* $(n_{i}^{\varepsilon})_{\varepsilon >0, \ i \in \left \lbrace 1,2 \right \rbrace}$ *converges in the sense of measures to* $n_{i}$. *Moreover*, *we have*
  $$ \mathrm{supp} \ \ n_{i}( \cdot , t) \subset \left \lbrace u( \cdot , t) = 0 \right \rbrace . $$

### Outline of the Paper

In Sect. 2, we detail the general approach and state the main technical results that lead to the proof of Theorem 1.2. Section 3 is devoted to the proofs of these technical results. In Sect. 4, we prove Theorem 1.2. Next, in Sect. 5, we detail the biological context that motivates our theoretical study. Finally, in Sect. 6, we illustrate our theoretical study by some numerical simulations in the framework given by our biological motivations. We also investigate numerically some open questions.

**Notations:** All along the paper, we adopt the following conventions:

- the letters $i,j$ refer, when there is no confusion possible, to an index in $\left \lbrace 1,2 \right \rbrace $,
- if $i$ and $j$ are used in a same equation then $i \neq j$,
- the bold mathematical characters are strictly reserved for vectors of $\mathbb{R}^{2}$ or matrix of $\mathcal{M}_{2}(\mathbb{R})$,
- the constants $c, C$ are taken positive and may change from line to line when there is no confusion possible (the capital letter is preferentially used for large constants and the small letter for small constants).

## The Hamilton Jacobi Approach

We develop in this part of the work a general approach for non-local cooperative systems. For technical reasons, we focus on a model with only two species and a uni-dimensional space. This part is largely inspired by [7] and [8], but since we study a coupled system, we cannot use the same arguments straight away. Unlike in the articles [5] and [8] for the single species problem, it is not possible to obtain directly a uniform BV estimate for the total mass $N_{\varepsilon}(t)$. There are additional mixing terms and, *a priori*, nothing prevents them to blow-up when $\varepsilon $ goes to 0. Moreover, one can not apply directly the method of [7] because the non-local total mass does not prevent the logarithm of the solution to be positive. We will circumvent these issues by employing a combination of the two former approaches.

### The Approach

Before dealing with the mathematical details, we propose an overview of the classical methods to treat this kind of problem as well as a presentation of heuristic arguments.

A local version of (1.6) was studied in [7] (i.e. with $N_{\varepsilon}$ replaced by $(n_{i}^{\varepsilon})_{i \in \left \lbrace 1,2 \right \rbrace}$). Moreover, the authors focus on general systems with more that two equations. We do not obtain the same level of generality than [7]. As we will see later, the hypothesis of having only two equations (rather than several) is a key hypothesis in our work. From a technical point of view, Barles, Evans and Souganidis do not prove any regularity results on $u_{i}^{\varepsilon}$ but they study the system through the semi-relaxed limit method by defining

$$ u_{*} (x,t) = \underset{i \in \left \lbrace 1,2 \right \rbrace}{\min}( \underset{(y,s) \to (x,t)}{\underset{\varepsilon \to 0}{\liminf} } \ u_{i}^{\varepsilon}(y,s)) \qquad \text{ and } \qquad u^{*}(x,t) = \underset{i \in \left \lbrace 1,2 \right \rbrace}{\max}( \underset{(y,s) \to (x,t)}{\underset{\varepsilon \to 0}{\limsup} } \ u_{i}^{\varepsilon}(y,s)). $$

We did not succeed in adapting this idea without proving any regularity results on $u_{i}^{\varepsilon}$. Indeed, with the semi-relaxed limit approach, one key point is to prove that $u^{*} \leq 0$. In [7], this claim is true; otherwise, it would be in contradiction with some natural bounds on $n_{\varepsilon}$ (obtained with the maximum principle). However, in our setting without any regularity result in space on $u_{i}^{\varepsilon}$, even if we have natural bounds on the total mass $N_{\varepsilon}$, nothing prevents the solution $u^{*}$ to be positive at a singular point. Indeed, contrary to the problem studied in [7], $u_{i}^{\varepsilon}$ may be positive on a sequence of intervals $I_{\varepsilon}$ with $\lambda (I_{\varepsilon}) \to 0$ (where $\lambda $ stands for the Lebesgue measure).

Therefore, we state regularity results in space on $u_{i}^{\varepsilon}$. Our result generalizes the case of the single population equation (1.2) (*i.e.* $\delta _{i} = 0$ and $n_{2} = 0$). In the first works treating this equation [5, 6, 8], the main result on the convergence of $u_{\varepsilon}$ was obtained by proving some BV-estimates on $N_{\varepsilon}$ and some bounds on $|\partial _{x} u_{1}^{\varepsilon}|$ by using the Bernstein method. Then obtaining the Lipschitz regularity of $u_{i}^{\varepsilon}$ with respect to time leads to the convergence by using the Arzela-Ascoli Theorem. Before, dealing with the Hamilton-Jacobi equation (1.13), we prove the convergence of $N_{\varepsilon}$ toward subsequence. We adapt the proof of [6] (Theorem 3.1) and [8] (Theorem 2.4). The proof of the Theorem 3.1 of [6] involves the positiveness of $r^{2}$ (equation (3.5) of [6]). In our work, it is not clear in general that

$$ 0 \leq \begin{pmatrix} 1 & 1 \end{pmatrix} \textbf{R}^{2} \begin{pmatrix} n_{1} \\ n_{2} \end{pmatrix} , $$

the right-hand side being what we would obtain in place of $r^{2}$.

To tackle this issue, we propose a precise estimate of $\frac{n_{1}^{\varepsilon}}{n_{2}^{\varepsilon}}$. Indeed, this estimate ensures that the exponential term is bounded and then one can apply the classical Bernstein method to obtain regularity in space. From this space regularity, we will deduce that $u_{i}^{\varepsilon}$ is Lipschitz with respect to time. Finally from this last result, we deduce that the family $N_{\varepsilon}$ converges. It will allow us to conclude.

We underline that the estimate of $\frac{n_{1}^{\varepsilon}}{n_{2}^{\varepsilon}}$ plays a similar role than the Harnack estimates obtained in [29, 36] in elliptic settings.

We formally write a Taylor expansion of $u_{i}^{\varepsilon}$:

$$ u_{i}^{\varepsilon}= u_{i} + \varepsilon v_{i} + o(\varepsilon ).$$

We first expect that $u_{1} = u_{2} = u$ since we do not expect a blow up of the exponential term. Next, by subtracting the two equations of (1.9) and using the fact that $u_{1} = u_{2}$, we obtain

$$ \begin{aligned} \big[(d_{1} - d_{2})(\partial _{x} u)^{2} + (r_{1} - r_{2} ) \big] \frac{n_{1}}{n_{2}} + \delta _{1} - \delta _{2} \left ( \frac{n_{1}}{n_{2}}\right )^{2} &= \varepsilon \big(\partial _{t} (v_{1} - v_{2}) + d_{1}[\partial _{x} v_{1}]^{2} - d_{2}[\partial _{x} v_{2}]^{2} \\ &\qquad{} - 2\partial _{x} u \partial _{x}(d_{1}v_{1} - d_{2}v_{2}) +o( \varepsilon ) \big). \end{aligned} $$

Taking formally, the limit $\varepsilon \to 0$, we expect

$$ \frac{n_{1} }{n_{2}} \sim \frac{[(d_{1} - d_{2})(\partial _{x} u)^{2} + (r_{1} - r_{2} ) ] + \sqrt{[(d_{1} - d_{2})(\partial _{x} u)^{2} + (r_{1} - r_{2} ) ]^{2} + 4\delta _{1}\delta _{2}} }{2 \delta _{2}}. $$

The above expression involves $\partial _{x} u$ which is not clearly defined yet. Notice here that in the special case $d_{1} = d_{2}$ the formula is simpler since the right-hand part is only defined thanks to the functions $r_{1},r_{2},\delta _{1},\delta _{2}$ and we expect

$$ \frac{n_{1} }{n_{2}} \sim \frac{ (r_{1} - r_{2} ) + \sqrt{ (r_{1} - r_{2} )^{2} + 4\delta _{1}\delta _{2}} }{2 \delta _{2}}.$$

#### Definition 2.1

Let $q_{i}$ be the unique positive root of

$$ P_{d_{i}, d_{j}}(X) = ([d_{i} -d_{j}](\partial _{x} u_{j}^{\varepsilon})^{2} + r_{i} -r_{j})X+ \delta _{i} - \delta _{j} X^{2},$$

2.1 $$ \text{ i.e. } q_{i} = \frac{ ([d_{i} - d_{j} ] (\partial _{x} u_{j}^{\varepsilon})^{2} + r_{i} - r_{j}) + \sqrt{([d_{i} - d_{j} ] (\partial _{x} u_{j}^{\varepsilon})^{2} + r_{i} - r_{j})^{2} + 4 \delta _{i} \delta _{j}}}{2 \delta _{j} }. $$

The fact that $\mathrm{deg}(P_{d_{i}, d_{j}}) = 2$ is important because it allows us to make a reasoning on the sign of $P_{d_{i}, d_{j}}$. Next, with this definition, we state the main technical statements that are necessary to prove Theorem 1.2.

#### Theorem 2.2

*Under the hypotheses* (H1), (H2), (H3) *and* (H4), *the following assertions hold true*.

1. Bounds. *There exists* $a',A',b,B$ *such that*
  2.2 $$ -bt - a'x^{2} -c \leq u_{i}^{\varepsilon}(x,t)\leq Bt - A'x + C. $$
2. Space regularity. *For any times* $0< t_{1}< T$ *and* $R>0$ *there exists a constant* $C_{t_{1}, T, R}>0$ *such that*
  2.3 $$ \underset{(x,t) \in [0, R] \times [t_{1}, T]}{\max}|\partial _{x} u_{i}^{\varepsilon}(x,t)| \leq C_{t_{1}, T, R}. $$
3. Ratio $\frac{n_{1}}{n_{2}}$. *For any positive time*, *we have*
  2.4 $$ \begin{aligned} &\varepsilon \left [ \ln (q_{1}(x, t)) - \frac{\varepsilon ^{4}}{t} \right ] \leq u_{1}^{\varepsilon}(x,t) - u_{2}^{\varepsilon}(x,t) \leq \varepsilon \left [ \ln (q_{1}(x, t)) + \frac{\varepsilon ^{4} }{t} \right ] \\ \textit{ and } \qquad & \varepsilon \left [ \ln (q_{2}(x,t)) - \frac{\varepsilon ^{4}}{t} \right ] \leq u_{2}^{\varepsilon}(x,t) - u_{1}^{\varepsilon}(x,t) \leq \varepsilon \left [ \ln (q_{2}(x,t)) + \frac{\varepsilon ^{4} }{t} \right ]. \end{aligned} $$
4. Time regularity. *The family* $(u_{i}^{\varepsilon})_{\varepsilon >0 , i\in \left \lbrace 1, 2 \right \rbrace}$ *is locally uniformly continuous with respect to time*.

Remark that the third item (the ratio estimates) comes after the space regularity result since when $d_{1}< d_{2}$, if $\partial _{x} u_{i}^{\varepsilon}$ is not locally bounded with respect to $\varepsilon $, one can not conclude the proof of (2.4). However, to prove the space regularity result, one needs an estimate similar to (2.4). We prove a weaker version of (2.4) as an intermediate result but we state only the stronger result in the theorem above. We also highlight that the terms $\varepsilon ^{4}$ has an exponent 4 that will be used in the proof of point 1. of Theorem 1.2.

### The Special Case $d_{1} = d_{2} = 1$

In this special setting, note that $q_{i}$ does not involve $\partial _{x} u_{j}^{\varepsilon}$ anymore. Therefore, the point 3. of Theorem 2.2 can be obtained directly by observing that

$$ - C(x+1) \leq \ln (q(x)) \leq C (x+1)$$

(for some large constant $C>0$). We refer to the forthcoming proof of Lemma 3.3 for more details. It follows that the point 2. of Theorem 2.2 can be obtained from the point 3.

Last, we can also derive formally a simpler equivalent equation for system (5.10) in the long time limit. We can assume $n_{1,\infty}(x)\simeq q(x)n_{2,\infty}(x)$ when $t\to +\infty $ and thus the quantity

$$ w(x) = n_{1,\infty}(x)+ n_{2,\infty}(x) = n_{2,\infty}(x)(1+q(x))$$

should satisfy the equation

2.5 $$ - \varepsilon ^{2} \dfrac{\partial ^{2} w}{\partial x^{2}}(x) = w(x) \big(r_{\infty}(x) - N(t) \big), $$

with $N(t) =\int _{0}^{+\infty}w(x,t)dx$ and where the global fitness function $r_{\infty}$ of the system writes

$$ r_{\infty}(x) = \dfrac{q(x)}{1+q(x)} \left ( r_{1}(x) + \delta _{2}(x) \right ) + \dfrac{1}{1+q(x)}(r_{2}(x)+\delta _{1}(x)). $$

Equation (2.5) is well understood. It is proved in [1, 31] that for each $\varepsilon $ there exists a unique solution which is the ground state of the Schroedinger operator

$$ \hat{H}:= - \varepsilon ^{2} \Delta - r_{\infty}. $$

First, we remark that

$$ q = \frac{[r_{1} - r_{2}] + \sqrt{[r_{1} - r_{2}]^{2} + 4 \delta _{1} \delta _{2} }}{2 \delta _{2}} \quad \text{ and } \quad q^{-1} = \frac{[r_{2} - r_{1}] + \sqrt{[r_{2} - r_{1}]^{2} + 4 \delta _{1} \delta _{2} }}{2 \delta _{1}}.$$

Recalling the definition (1.11), we notice that

$$ r_{H}^{I_{2}} = \delta _{2} q + r_{2} = \delta _{1} q^{-1} + r_{1}.$$

We conclude

2.6 $$ r_{\infty}= \dfrac{1}{1+q}\big( q[r_{1} + q^{-1}\delta _{1}] + [r_{2} + q \delta _{2}] \big) = r_{H}^{I_{2}}(x). $$

Hence, the Hamiltonian fitness referred to above describes the behaviour of the system in both the limits $\varepsilon \to 0$ and $t\to +\infty $. This function is formally the equivalent fitness of the overall system formed by the two cooperating populations. They adjust their fitness parameter $x$ in function of the maximum points of $r_{H}(x)$.

## The Intermediate Technical Results

Here, we prove all the statements of Theorem 2.2 and some intermediate results that are not stated above.

### Bounds on $u_{i}^{\varepsilon}$

First, we focus on the bounds for $u_{i}^{\varepsilon}$. The method is quite standard (see e.g. [4] for a general introduction of this kind of method or [8] for a closer use of this method) but some new difficulties arise from the interplay between the two populations.

#### Proof of 1. of 2.2

We split the proof into two parts: the upper bound and then the lower one.

**The upper bound.** First, we define $\psi = - A'x+ Bt + C$ with $A',B>0$ and $A' < A$ that will be fixed later on. We also introduce $w^{\varepsilon}= \max (u_{1}^{\varepsilon}, u_{2}^{\varepsilon})$ and $i \in \left \lbrace 1,2 \right \rbrace $ the corresponding integer. From assumption (H4), it is clear that $w^{\varepsilon}(t= 0) \leq \psi (x, t= 0)$. Next, we consider

$$ T := \inf \left \lbrace t>0: \quad \exists x > 0, \ w^{\varepsilon}(x,t) > \psi (x,t) \right \rbrace .$$

We prove by contradiction that $T = +\infty $. Assume $T < +\infty $. We distinguish two cases:

- **Case 1:** *There exists* $x_{0}>0$ *such that* $w^{\varepsilon}(x_{0}, T) = \psi (x_{0}, T)$*.* It follows by definition of $T$
  $$ \partial _{t} (w^{\varepsilon}- \psi )(x_{0}, T) \geq 0, \quad - d_{i} \partial _{xx} (w^{\varepsilon}- \psi )(x_{0}, T) \geq 0 \quad \text{ and } $$
  $$ d_{i} \partial _{x} w^{\varepsilon}(x_{0}, T) = d_{i} \partial _{x} \psi (x_{0}, T). $$
  The definition of $w^{\varepsilon}$ yields that the exponential part is bounded by 1. From this bound and (H2), it follows
  $$\begin{aligned} B - d_{i} A^{\prime \,2} &\leq \left ( \partial _{t} \psi - \varepsilon d_{i} \partial _{xx} \psi - d_{i}( \partial _{x} \psi )^{2} \right ) (x_{0}, T) \\& \leq \left ( \partial _{t} w^{\varepsilon}- \varepsilon d_{i} \partial _{xx} w^{\varepsilon}- d_{i} ( \partial _{x} w^{\varepsilon})^{2} \right ) (x_{0}, T) \leq C_{R} + C_{\delta} \end{aligned}$$
  which is impossible for $B > C_{R} + C_{\delta}+ d_{i} A^{\prime \,2}$. (We remark that $x_{0}>0$ according to the Neumann boundary conditions imposed on $w^{\varepsilon}$. Moreover, the first inequality above is a strict inequality whenever $d_{i} = 0$.)
- **Case 2:** *There holds* $\inf (w^{\varepsilon}- \psi )(\cdot , T) = 0$ *with* $w^{\varepsilon}(\cdot , T) < \psi (\cdot , T)$*.* In this case, we introduce $\psi _{\gamma}:= \psi (x,t) - \gamma e^{-\frac{\varepsilon}{t}}$ with $\gamma \in (0, 1]$ and
  $$ T_{\gamma}:= \inf \left \lbrace t>0: \quad \exists x > 0, \ w^{\varepsilon}(x,t) > \psi _{\gamma}(x,t) \right \rbrace . $$
  Since $\psi _{\gamma}(t= 0^{+}) = \psi (t= 0)$ and $\psi _{\gamma}(T) < \psi (T)$, we have $0< T_{\gamma}\leq T$. Remark also that $T_{1} \leq T_{\gamma}$ for $\gamma < 1$. Moreover, since $\partial _{t} \psi _{\gamma}(\cdot , t) = B - \frac{\gamma e^{-\frac{\varepsilon}{t}}}{t^{2}} < \partial _{t} \psi ( \cdot , t)$, we conclude as in case 1 that $w^{\varepsilon}(\cdot , T_{\gamma})< \psi _{\gamma}(\cdot , T_{\gamma})$. We claim that $T_{\gamma}< T$. Indeed, since there exists, by definition of $T$, a sequence $x_{n}>0$ such that $(w^{\varepsilon}- \psi )(x_{n} , T) \to 0$. If $T = T_{\gamma}$, it would imply for $n \in \mathbb{N} $ large enough that $(w^{\varepsilon}- \psi )(x_{n} , T) < \frac{\gamma e^{\frac{\varepsilon}{T}}}{2}$ and a contradiction follows from
  $$ 0 < (w^{\varepsilon}- \psi _{\gamma})(x_{n} , T) < - \frac{\gamma e^{\frac{\varepsilon}{T}}}{2}.$$
  We deduce the existence of $\tau \in \, ]T_{\gamma}, T[$ and $x_{\tau}>0$ such that
  $$ \psi _{\gamma}(x_{\tau}, \tau ) < w^{\varepsilon}(x_{\tau}, \tau ) . $$
  Finally, we introduce
  $$ \begin{aligned} &\psi _{\gamma , \sigma} := \psi _{\gamma}+ \sigma (x- x_{\tau})^{2} \\ \text{and } \quad & T_{\sigma}:= \inf \left \lbrace t>0: \quad \exists x > 0, \ w^{\varepsilon}(x,t) > \psi _{\gamma , \tau}(x,t) \right \rbrace . \end{aligned} $$
  We underline that $T_{1} \leq T_{\gamma}\leq T_{\sigma}\leq \tau < T $ since $\psi _{\gamma , \sigma}(x_{\tau},\tau )< w^{\varepsilon}(x_{\tau}, \tau ) $.
  Moreover, for all $x>0$ such that $|x - x_{\tau}| > \sqrt{\frac{\gamma e^{-\frac{1}{T_{\sigma}}}}{\sigma}}$, one has that $w^{\varepsilon}(x, T_{\sigma}) \leq \psi (x, T_{\sigma})\leq \psi _{ \gamma , \sigma} (x , T_{\sigma})$ since $T_{\sigma}< T$. We deduce that there exists $x_{0} \in B(x_{\tau},\sqrt{ \frac{\gamma e^{-\frac{1}{T_{\sigma}}}}{\sigma}}) $ such that
  $$ 0 =( w^{\varepsilon}- \psi _{\gamma , \sigma} )(x_{0}, T_{\sigma}) = \max ( w^{\varepsilon}- \psi _{\gamma , \sigma} ).$$
  As above, we deduce that
  $$ B -\frac{d_{i}\gamma e^{-\frac{1}{T_{\sigma}}}}{T_{\sigma}^{2}} - d_{i}[A' + 2\sigma (x_{0} - x_{\tau})]^{2} - \varepsilon 2 \sigma \leq C_{R} + C_{\delta}.$$
  Next, using the bounds on $|x_{0} - x_{\tau}|$ and $T_{\sigma}$, we conclude that
  $$\begin{aligned} B -\frac{d_{i}\gamma}{T_{1}^{2}} -d_{i} [A' + 2\sqrt{\gamma \sigma}]^{2} - \varepsilon d_{i} 2 \sigma & \leq B - \frac{d_{i}\gamma e^{-\frac{1}{T_{\sigma}}}}{T_{\sigma}^{2}} - d_{i}[A' + 2\sigma (x_{0} - x_{\tau})]^{2} - \varepsilon d_{i} 2 \sigma \\& \leq C_{R} + C_{\delta}. \end{aligned}$$
  Passing to the inferior limits $\sigma \to 0$ and then $\gamma \to 0$, it follows
  $$ B - d_{i} A^{\prime \,2} \leq C_{R} + C_{\delta}$$
  which is absurd for $B> C_{R} + C_{\delta}+ d_{i} A^{\prime \,2}$.

It concludes the proof of the upper bound.

**The lower bound.** First, we define $\phi (x,t) = - a'x^{2}-bt - c$ with $a',b>0$ two free parameters satisfying $a < a'$. We prove the lower bound for $u^{\varepsilon}_{i}$ with $i \in \left \lbrace 1,2 \right \rbrace $. As above, we introduce

$$ T := \inf \left \lbrace t>0 : \quad \exists x>0 , \ u_{i}^{\varepsilon}(x,t) < \phi (x,t) \right \rbrace .$$

Remarking that $\phi (x, t=0) \leq u^{\varepsilon}_{i}(x,t=0)$, we deduce that $T>0$. As for the upper bound, we distinguish the proof into two cases:

- **Case 1:** *There exists* $x_{0} >0$ *such that* $u^{\varepsilon}_{i}(x_{0}, T) = \phi (x_{0}, T)$*.* In this case, we have
  $$ \partial _{t} (u_{1}^{\varepsilon}- \phi )(x_{0}, T) \leq 0, \quad - \partial _{xx} (u_{1}^{\varepsilon}- \phi )(x_{0}, T) \leq 0 \quad \text{ and } \quad \partial _{x} (u_{1}^{\varepsilon}- \phi )(x_{0}, T) = 0.$$
  We deduce that
  $$\begin{aligned} -b -d_{i}(2a'x_{0})^{2} &\leq \left (\partial _{t} \phi - \varepsilon d_{i} \partial _{xx} \phi - d_{i} | \partial _{x} \phi |^{2} \right )(x_{0}, T) \\&\geq \left (\partial _{t} u_{1}^{\varepsilon}- \varepsilon d_{i} \partial _{xx} u_{1}^{\varepsilon}- d_{i} |\partial _{x} u_{1}^{\varepsilon}|^{2} \right )(x_{0}, T) \geq - C_{R}. \end{aligned}$$
  It is impossible for $a',b$ large enough (the first above inequality is strict if and only if $d_{i} = 0$).
- **Case 2:** *There holds* $u_{i}^{\varepsilon}(x, T) > \phi (x, T)$ *for all* $x>0$*.* As for the upper bound, we introduce for $\gamma \in (0, 1]$
  $$ \begin{aligned} &\phi _{\gamma}= \phi + \gamma e^{-\frac{\varepsilon}{t}} \\ \text{ and } \quad & T_{\gamma}:=\inf \left \lbrace t>0 : \quad \exists x>0 , \ u_{1}^{\varepsilon}(x,t) < \phi _{\gamma}(x,t) \right \rbrace . \end{aligned} $$
  It is clear that $0 < T_{1} \leq T_{\gamma}< T$. Next, there exists $\tau \in ]T_{\gamma}, T[$ and $x_{\tau}> 0$ such that
  $$ \phi _{\gamma}(x_{\tau}, \tau ) > u_{1}^{\varepsilon}(x_{\tau}, \tau ). $$
  We introduce
  $$ \begin{aligned} &\phi _{\gamma , \sigma} = \phi _{\gamma}- \sigma (x - x_{\tau})^{2} \\ \text{ and } \quad & T_{\sigma}:=\inf \left \lbrace t>0 : \quad \exists x>0 , \ u_{i}^{\varepsilon}(x,t) < \phi _{\gamma , \sigma}(x,t) \right \rbrace . \end{aligned} $$
  Moreover, we have $0 < T_{1} \leq T_{\gamma}< T_{\sigma}< \tau \leq T$. Since for $x \in B^{c}(x_{\tau}, \sqrt{\frac{\gamma}{\sigma}})$, we have $\phi _{\gamma , \sigma} (x, T_{\sigma})< \phi (x, T_{\sigma}) < u_{i}^{\varepsilon}(x, T_{\sigma}) $ (since $T_{\sigma}< \tau $), it follows the existence of $x_{0} \in B(x_{\tau}, \sqrt{\frac{\gamma}{\sigma}})$ such that
  $$ u_{i}^{\varepsilon}(x_{0}, T_{\sigma}) = \phi _{\gamma , \sigma} (x_{0}, T_{\sigma}).$$
  A direct computation implies
  $$ \begin{aligned} -b +\frac{d_{i} \gamma}{T_{1}^{2}} -d_{i} [2a'x_{0} - 2\sqrt{\gamma \sigma}]^{2} + \varepsilon d_{i} 2 (\sigma ) &\geq \left (\partial _{t} \phi - \varepsilon d_{i}\partial _{xx} \phi - d_{i}[\partial _{x} \phi ]^{2} \right ) (x_{0}, T) \\ &\geq \left (\partial _{t} u_{i}^{\varepsilon}- \varepsilon d_{i} \partial _{xx} u_{i}^{\varepsilon}- d_{i} [\partial _{x} u_{i}^{\varepsilon}]^{2} \right ) (x_{0}, T) \\ &\geq -C_{R} . \end{aligned} $$
  Taking the inferior limits $\sigma \to 0$ and $\gamma \to 0$ and $b$ large enough, leads to the desired contradiction.

It concludes the proof. □

### A First Weak Asymptotic Result for $u_{i}^{\varepsilon}- u_{j}^{\varepsilon}$

As mentioned in the comment that follows the statement of Theorem 2.2, we only prove a first imprecise (but necessary) result on $u_{i}^{\varepsilon}- u_{j}^{\varepsilon}$. For this purpose, we introduce

#### Definition 3.1

Let $q_{i}^{+}$ be defined by

3.1 $$ q_{i}^{+} =\left \lbrace \begin{aligned} & \frac{ \sqrt{([d_{i} - d_{j} ] (\partial _{x} u_{j}^{\varepsilon})^{2} + r_{i} - r_{j})^{2} + 4 \delta _{i} \delta _{j}}}{2 \delta _{j} } && \quad \text{ when } d_{i} < d_{j}, \\ &q_{i} && \quad \text{ when } d_{i} \geq d_{j}. \end{aligned} \right . $$

We emphasize that

3.2 $$ q_{i}(x,t) \leq q_{i}^{+}(x,t) \qquad \forall (x,t) \in \mathbb{R}^{+} \times \mathbb{R}^{+}. $$

This new quantity is introduced in order to prove the following result

#### Lemma 3.2

*Under the hypothesis* (H2), *we have*

$$ - c (x+1)\leq \ln (q_{1}^{+}(x,t)).$$

#### Proof

By definition of $q_{1}^{+}$, in any case and for all $\varepsilon >0$, we have

$$ \frac{\ln (\delta _{1}(x))-\ln (\delta _{2}(x))}{2} \leq \ln \left ( \sqrt{\frac{\delta _{1}(x)}{\delta _{2}(x)}}\right ) \leq \ln ( q_{1}^{+}(x,t) ) . $$

Thanks to (H2), we have

$$ -C_{\delta}x - \ln (C_{\delta}) \leq \ln ( q_{1}^{+}(x,t) ). $$

 □

Notice that when $d_{1}< d_{2}$, the conclusion of Lemma 3.2 may be false for $q$ if $|\partial _{x} u_{2}^{\varepsilon}|$ is not locally bounded. With this result, one can state the following lemma:

#### Lemma 3.3

*Under the hypothesis* (H1), (H2), (H3) *and* (H4), *we have*

$$ \varepsilon \left ( \ln (q_{2}^{+}(x,t)) - \frac{\varepsilon ^{4} }{t} \right ) \leq (u_{1}^{\varepsilon}- u_{2}^{\varepsilon})(x,t) \leq \varepsilon \left ( \ln (q_{1}^{+}(x,t)) + \frac{\varepsilon ^{4} }{t} \right ) . $$

#### Proof

Set $(x_{0}, t_{0}) \in \mathbb{R}^{+}\times \mathbb{R}^{+}$, $\varepsilon >0$ and $\mu >0$. The aim is to prove that

$$ \varepsilon \left ( \ln (q_{2}^{+}(x_{0},t_{0})) - \frac{\varepsilon ^{4} }{t_{0}} \right ) \leq (u_{1}^{\varepsilon}- u_{2}^{\varepsilon})(x_{0},t_{0}) \leq \varepsilon \left ( \ln (q_{1}^{+}(x_{0},t_{0})) + \frac{\varepsilon ^{4} }{t_{0}} \right ) . $$

First, we assume $d_{1}>0$. We introduce

$$\begin{aligned} \tau _{\mu}:= \inf \bigg\lbrace & t > 0: \quad \exists x>0 \quad \text{ such that } \quad\\ & (u_{1}^{\varepsilon}- u_{2}^{\varepsilon})(x,t) - \mu ^{-1} (x-x_{0})^{2} > \varepsilon \left [ \ln (q_{1}^{+}(x,t)) + \frac{\varepsilon ^{4}}{t}\right ] \bigg\rbrace . \end{aligned}$$

Thanks to 1. of Theorem 2.2, we have $\tau _{\mu}>0$. Remark also that for all $\mu <1$, we have $\forall (x,t) \in \mathbb{R}^{+} \times [0, \tau _{1}]$

$$\begin{aligned} (u_{1}^{\varepsilon}- u_{2}^{\varepsilon})(x,t) - \mu ^{-1} (x-x_{0})^{2} & = (u_{1}^{\varepsilon}- u_{2}^{\varepsilon})(x,t) - (x-x_{0})^{2} + [ \mu ^{-1}-1] (x-x_{0})^{2}\\ &< \varepsilon \left [ \ln q_{1}(x,t) + \frac{\varepsilon ^{4}}{t} \right ] . \end{aligned}$$

It follows that $\tau _{\mu}>\tau _{1}>0$ for all $\mu <1$. Next, we distinguish two cases:

1. $\underset{\mu \rightarrow 0}{\limsup} \ \tau _{\mu}> t_{0}$,
2. $\underset{\mu \rightarrow 0}{\liminf} \ \tau _{\mu}\leq t_{0}$.

We will only consider the second case, since it is clear that in the first case the conclusion holds true.

We prove by contradiction that this case can not hold. Let $\mu _{n} \to 0$ be such that $\tau _{\mu _{n}}$ converges to $\underset{\mu \rightarrow 0}{\liminf} \tau _{\mu}$. For sake of readability, we replace $\tau _{\mu _{n}}$ by $\tau _{\mu}$. Notice that this limit belongs to $[\tau _{1}, t_{0}]$.

According to the point 1. of Theorem 2.2 and Lemma 3.2, we have

$$ (u_{1}^{\varepsilon}- u_{2}^{\varepsilon})(x, \tau _{\mu}) -\mu ^{-1}(x-x_{0})^{2} - \varepsilon \left [\ln (q_{1}^{+}(x,\tau _{\mu})) + \frac{\varepsilon ^{4}}{\tau _{\mu}} \right ] \underset{ x \to +\infty}{\longrightarrow} -\infty .$$

It follows the existence of $x_{\mu}>0$ such that

$$ \begin{aligned} 0 \ =& \ (u_{1}^{\varepsilon}- u_{2}^{\varepsilon})(x_{\mu}, \tau _{\mu}) - \mu ^{-1}(x_{\mu}-x_{0})^{2}- \varepsilon \left [ \ln (q_{1}^{+}(x_{\mu}, \tau _{\mu})) + \frac{\varepsilon ^{4}}{\tau _{\mu}} \right ] \\ =& \ \underset{ x >0}{\max} \ (u_{1}^{\varepsilon}- u_{2}^{\varepsilon})(x, \tau _{\mu}) - \mu ^{-1} (x-x_{0})^{2} - \varepsilon \left [ \ln (q_{1}^{+}(x,\tau _{\mu})) + \frac{\varepsilon ^{4}}{\tau _{\mu}} \right ]. \end{aligned} $$

Moreover, we observe that as $\mu \to 0$, one has $x_{\mu}\to x_{0}$. One also has

3.3 $$ \begin{aligned} &\partial _{t} (u_{1}^{\varepsilon}- u_{2}^{\varepsilon})(x_{\mu}, \tau _{\mu}) = -\frac{\varepsilon ^{5}}{\tau _{\mu}^{2}} + \varepsilon \partial _{t} \left (\ln (q_{1}^{+}(x_{\mu}, \tau _{\mu}) \right ), \\ &\partial _{x}(u_{1}^{\varepsilon}- u_{2}^{\varepsilon})(x_{\mu}, \tau _{\mu}) = 2\mu ^{-1} (x_{\mu}- x_{0}) + \varepsilon \partial _{x} ( \ln (q_{1}^{+}(x_{\mu}, \tau _{\mu})) \\ \text{ and } \qquad -&\partial _{xx} (u_{1}^{\varepsilon}- u_{2}^{\varepsilon})(x_{\mu}, \tau _{\mu}) \geq 2\mu ^{-1} + \varepsilon ( \partial _{xx}\ln (q_{1}^{+}(x_{\mu}, \tau _{\mu})). \end{aligned} $$

Since $(x_{\mu}, \tau _{\mu})$ converges as $\mu \to 0$ and all the involved functions are continuous, we deduce the existence of $C>0$ (independent of $\mu $ but that may depend on $\varepsilon $) such that for all $\mu >0$ small enough,

3.4 $$ \begin{aligned} \max \Big(&| \frac{\varepsilon ^{5}}{\tau _{\mu}^{2}} + \varepsilon \partial _{t} \left (\ln (q_{1}^{+}(x_{\mu}, \tau _{\mu}) \right ) |, | \partial _{x} ( \ln (q_{1}^{+}(x_{\mu}, \tau _{\mu}))| , \\ & \qquad | \partial _{xx}( \ln (q_{1}^{+}(x_{\mu}, \tau _{\mu}))| ,| \partial _{x} (u_{1}^{\varepsilon}+ u_{2}^{\varepsilon}) (x_{\mu}, t_{\mu})|, |\partial _{xx} u_{2}^{\varepsilon}(x_{\mu}, \tau _{\mu})|\Big) < C. \end{aligned} $$

We subtract the equations for $u_{1}^{\varepsilon}$ and $u_{2}^{\varepsilon}$ and we obtain

3.5 $$ \begin{aligned} &\partial _{t} (u_{1}^{\varepsilon}- u_{2}^{\varepsilon}) - d_{1} \partial _{x}(u_{1}^{\varepsilon}- u_{2}^{\varepsilon}) \partial _{x}(u_{1}^{\varepsilon}+ u_{2}^{\varepsilon}) - d_{1}\varepsilon \partial _{xx} (u_{1}^{\varepsilon}- u_{2}^{\varepsilon}) - [d_{1} + d_{2}] \varepsilon \partial _{xx} u_{2}^{\varepsilon}\\ = \quad & [d_{1} - d_{2}] (\partial _{x} u_{2}^{\varepsilon})^{2} + r_{1} - r_{2} + \delta _{1} e^{ \frac{u_{2}^{\varepsilon}- u_{1}^{\varepsilon}}{\varepsilon} }- \delta _{2} e^{\frac{u_{1}^{\varepsilon}- u_{2}^{\varepsilon}}{\varepsilon} } \\ = \quad & e^{ \frac{u_{2}^{\varepsilon}- u_{1}^{\varepsilon}}{\varepsilon }} P_{d_{1},d_{2}}(e^{ \frac{u_{1}^{\varepsilon}- u_{2}^{\varepsilon}}{\varepsilon}}). \end{aligned} $$

Next, we evaluate the above equation at $(x_{\mu}, \tau _{\mu})$. First, since $(u_{1}^{\varepsilon}- u_{2}^{\varepsilon})(x_{\mu}, \tau _{\mu}) = \varepsilon \ln (q_{1}^{+}(x_{\mu}, \tau _{\mu})) + \mu ^{-1}(x_{\mu}-x_{0})^{2} +\frac{ \varepsilon ^{4}}{\tau _{\mu}}$, we deduce that

$$ q_{1}(x_{\mu}, \tau _{\mu}) \leq q_{1}^{+}(x_{\mu}, \tau _{\mu}) < e^{ \frac{(u_{1}^{\varepsilon}- u_{2}^{\varepsilon})(x_{\mu}, \tau _{\mu})}{\varepsilon}}.$$

It follows

$$ \left ( e^{\frac{u_{2}^{\varepsilon}- u_{1}^{\varepsilon}}{\varepsilon }} P_{d_{1},d_{2}}(e^{ \frac{u_{1}^{\varepsilon}- u_{2}^{\varepsilon}}{\varepsilon}}) \right ) (x_{\mu}, \tau _{\mu}) \leq 0. $$

We deduce thanks to (3.3), (3.4) and (3.5) that there holds for $\mu $ small enough

$$\begin{aligned} 0&< \quad C + C[2\mu ^{-1} |x_{\mu}- x_{0}| + C ] + 2 \varepsilon \mu ^{-1} + \varepsilon C (1 + d_{1} + d_{2}) \\ &=\quad \left (\partial _{t} (u_{1}^{\varepsilon}- u_{2}^{\varepsilon}) - d_{1} \partial _{x}(u_{1}^{\varepsilon}- u_{2}^{\varepsilon}) \partial _{x}(u_{1}^{\varepsilon}+ u_{2}^{\varepsilon}) - \varepsilon d_{1} \partial _{xx} (u_{1}^{\varepsilon}- u_{2}^{\varepsilon}) \right ) (x_{\mu}, t_{\mu}) \\ &\qquad{}- [d_{1} + d_{2}] \varepsilon \partial _{xx} u_{2}^{\varepsilon}(x_{\mu}, \tau _{\mu}) \\ &= \quad \left ( e^{ \frac{u_{2}^{\varepsilon}- u_{1}^{\varepsilon}}{\varepsilon }} P_{d_{1}, d_{2}}(e^{ \frac{u_{1}^{\varepsilon}- u_{2}^{\varepsilon}}{\varepsilon}}) \right ) (x_{\mu}, \tau _{\mu}) \\ &\leq \quad 0. \end{aligned}$$

We have reached the desired contradiction.

If $d_{1} = 0$, we provide the same arguments with

$$\begin{aligned} \tau _{\mu}:= \inf \bigg\lbrace & t > 0: \quad \exists x>0 \quad \text{ s.t. } \quad \\ & (u_{1}^{\varepsilon}- u_{2}^{\varepsilon})(x,t) - \mu ^{-1} (x-x_{0})^{2} > \varepsilon \left [ \ln (q_{1}^{+}(x,t)) + \frac{\varepsilon ^{4}}{t}\right ] + \mu [t- t_{0}] \bigg\rbrace . \end{aligned}$$

The proof for $q_{2}^{+}$ is identical by studying $u_{2}^{\varepsilon}- u_{1}^{\varepsilon}$. Therefore, we let it to the reader. □

It follows the following corollary

#### Corollary 3.4

*Under the hypothesis* (H1), (H2), (H4) *and* (H3) *we have*

3.6 $$ \frac{n_{i}^{\varepsilon}(x,t) }{n_{j}^{\varepsilon}(x,t)} = e^{\frac{(u_{i}^{\varepsilon}- u_{j}^{\varepsilon})(x,t)}{\varepsilon}} \leq C e^{C_{\delta}x + \frac{\varepsilon ^{3}}{t} } [(\partial _{x} u_{j}^{\varepsilon}(x,t))^{2} + 1] . $$

#### Proof

We focus on the case $i=1, \ j=2$, the other case works exactly the same. According to Lemma 3.3, it is sufficient to prove that

$$ q_{1}^{+} (x,t) \leq C e^{C_{\delta}x } [\partial _{x} u_{2}^{\varepsilon}(x,t)^{2} + 1] . $$

First, we remark that thanks to (H2)

$$ \frac{1}{\delta _{1}(x)} \leq Ce^{C_{\delta}x}. $$

Next, we treat the numerator of $q_{1}^{+}$. When $d_{1}< d_{2}$, we have

$$ \begin{aligned} \sqrt{\left ((d_{1}-d_{2}) (\partial _{x} u_{2}^{\varepsilon})^{2} + (r_{1}-r_{2}) \right )^{2} + 4 \delta _{1} \delta _{2} }&\leq (\partial _{x} u_{2}^{\varepsilon})^{2} \sqrt{\left ((d_{1}-d_{2}) + \frac{(r_{1}-r_{2})}{(\partial _{x} u_{2}^{\varepsilon})^{2}}\right )^{2} + \frac{4 \delta _{1} \delta _{2}}{(\partial _{x} u_{2}^{\varepsilon})^{4}} } \\ &\leq (\partial _{x} u_{2}^{\varepsilon})^{2} \sqrt{\left ((d_{1} +d_{2} + \frac{2 C_{R}}{(\partial _{x} u_{2}^{\varepsilon})^{2}}\right )^{2} + \frac{4 C_{\delta}^{2}}{(\partial _{x} u_{2}^{\varepsilon})^{4}} } \\ &\leq C[(\partial _{x} u_{2}^{\varepsilon})^{2}+ 1]. \end{aligned} $$

Combining the two above equations the conclusion follows.

For the case, $d_{1}\geq d_{2}$, we simply have thanks to the above computations

$$ q_{1}^{+} (x,t) \leq \left [(\partial _{x} u_{2})^{2} (C + \frac{(d_{1} -d_{2})}{2}+ 1) + C_{R} \right ] e^{C_{\delta}x } \leq C e^{C_{\delta}x} [(\partial _{x} u_{2}^{\varepsilon}(x,t)^{2} + 1] .$$

 □

### Space Regularity of $u_{i}^{\varepsilon}$

#### Proof of 2. of Theorem 2.2

First, we fix an initial time $t_{1}>0$ and a maximal time $T>0$ and a bound $R>0$. We focus our study in the set $(t_{1}, T) \times [0, R]$.

Next, we define $u_{i}^{\varepsilon}= f(v_{i}^{\varepsilon})$ where $v_{i}^{\varepsilon}$ will be chosen later on. A direct computation yields:

$$ \partial _{t} u_{i}^{\varepsilon}= f'(v_{i}^{\varepsilon}) \partial _{t} v_{i}^{\varepsilon}, \quad \partial _{x} u_{i}^{\varepsilon}= f'(v_{i}^{\varepsilon}) \partial _{x} v_{i}^{\varepsilon}\quad \text{ and } \quad \partial _{xx} u_{i}^{\varepsilon}= f'(v_{i}^{\varepsilon}) \partial _{xx} v_{i}^{\varepsilon}+ f''(v_{i}^{\varepsilon})[ \partial _{x} v_{i}^{\varepsilon}]^{2}.$$

Replacing in the $i$th equation (1.9), it follows

3.7 $$ \partial _{t} v_{i}^{\varepsilon}- \varepsilon d_{i} \partial _{xx} v_{i}^{\varepsilon}- d_{i} \left ( \varepsilon \frac{ f''(v_{i}^{\varepsilon})}{f'(v_{i}^{\varepsilon})} + f'(v_{i}^{\varepsilon}) \right ) [\partial _{x} v_{i}^{\varepsilon}]^{2}= \frac{r_{i}(x) - N(t) + \delta _{i}(x) e^{\frac{u_{j}^{\varepsilon}- u_{i}^{\varepsilon}}{\varepsilon}}}{f'(v_{i}^{\varepsilon})} .$$

Next, we differentiate (3.7) with respect to $x$ and we multiply by $\partial _{x} v_{i}^{\varepsilon}$ to obtain:

$$ \begin{aligned} &\partial _{t} ([\partial _{x} v_{i}^{\varepsilon}]^{2}) -\varepsilon d_{i} \partial _{xx} ([\partial _{x} v_{i}^{\varepsilon}]^{2}) + 2 \varepsilon d_{i} (\partial _{xx} v_{i}^{\varepsilon})^{2}-4 d_{i} \left ( \frac{f''(v_{i}^{\varepsilon})}{f'(v_{i}^{\varepsilon})} + f'(v_{i}^{\varepsilon}) \right ) \partial _{x}{ ([\partial _{x} v_{i}^{\varepsilon}]^{2})} \partial _{x} v_{i}^{\varepsilon}\\ &- 2d_{i}\left ( \varepsilon \frac{f'''(v_{i}^{\varepsilon})}{f'(v_{i}^{\varepsilon}) }- \varepsilon \frac{f''(v_{i}^{\varepsilon})^{2}}{f'(v_{i}^{\varepsilon})} + f''(v_{i}^{\varepsilon}) \right ) [\partial _{x} v_{i}^{\varepsilon}]^{4} =-2 \frac{f''(v_{i}^{\varepsilon})}{f'(v_{i}^{\varepsilon})}r_{i} \partial _{x} v_{i}^{\varepsilon}+2 \frac{\partial _{x} r_{i}}{f'(v_{i}^{\varepsilon})} \partial _{x} v_{i}^{\varepsilon}\\ & +2e^{ \frac{f(v_{j}^{\varepsilon}) - f(v_{i}^{\varepsilon}) }{\varepsilon}} \left ( \frac{\delta _{i}' f'(v_{i}^{\varepsilon}) - \delta _{i} f''(v_{i}^{\varepsilon}) }{f'(v_{i}^{\varepsilon})^{2}} \partial _{x} v_{i}^{\varepsilon}+ \delta _{i}\! \left [\! \frac{\partial _{x} v_{j}^{\varepsilon}f'(v_{j}^{\varepsilon}) \partial _{x} v_{i}^{\varepsilon}f'(v_{i}^{\varepsilon}) - (\partial _{x} v_{i}^{\varepsilon}f'(v_{i}^{\varepsilon}))^{2} }{\varepsilon f'(v_{i}^{\varepsilon})^{2}} \right ]\right )\!. \end{aligned} $$

Next, we assume that $(\partial _{x} u_{i}^{\varepsilon})^{2} = \max ( (\partial _{x} u_{1}^{\varepsilon})^{2}, (\partial _{x} u_{2}^{\varepsilon})^{2})$. It follows

$$ \frac{\partial _{x} v_{j}^{\varepsilon}f'(v_{j}^{\varepsilon}) \partial _{x} v_{i}^{\varepsilon}f'(v_{i}^{\varepsilon}) - (\partial _{x} v_{i}^{\varepsilon}f'(v_{i}^{\varepsilon}))^{2} }{\varepsilon f'(v_{i}^{\varepsilon})^{2}} \leq 0.$$

Next, by defining $f(v) = 2D - v^{2}$ where $D$ is large enough such that $f(v)>D$ (thanks to point 1. of Theorem 2.2) and dividing by $|\partial _{x} v_{i}^{\varepsilon}|$, we obtain thanks to Corollary 3.4

$$ \begin{aligned} &\partial _{t} (|\partial _{x} v_{i}^{\varepsilon}|) -\varepsilon d_{i} \partial _{xx} (|\partial _{x} v_{i}^{\varepsilon}|)-4 d_{i} \left ( \frac{f''(v_{i}^{\varepsilon})}{f'(v_{i}^{\varepsilon})} + f'(v_{i}^{\varepsilon}) \right ) \partial _{x}{ ([\partial _{x} v_{i}^{\varepsilon}]^{2})} \partial _{x} v_{i}^{\varepsilon}+4 d_{i} [ \partial _{x} v_{i}^{\varepsilon}]^{3} -4\frac{C_{r}}{D} \\ &\leq 2e^{ \frac{f(v_{j}^{\varepsilon}) - f(v_{i}^{\varepsilon}) }{\varepsilon}} \left ( \frac{\delta _{i}' f'(v_{i}^{\varepsilon}) - \delta _{i} f''(v_{i}^{\varepsilon}) }{f'(v_{i}^{\varepsilon})^{2}} \right ) \\ &\leq \frac{2Ce^{\frac{\varepsilon ^{4}}{t_{1}}}(|\partial _{x} u_{i}^{\varepsilon}|^{2} + 1)}{\delta _{i}} \max \left ( \frac{\delta _{i}' f'(v_{i}^{\varepsilon}) - \delta _{i} f''(v_{i}^{\varepsilon}) }{f'(v_{i}^{\varepsilon})^{2}} , 0\right ) . \end{aligned} $$

Next, thanks to Corollary 3.4, for any $R_{0}>0$, it follows the existence of a large constant $C(t_{1}, T)$ (independent of $\varepsilon $), such that for

$$ \theta (T, R_{0} ) = C(t_{1}, T) e^{C_{\delta}R_{0}} $$

we have

$$\begin{aligned} &\partial _{t} (|\partial _{x} v_{i}^{\varepsilon}|) -\varepsilon d_{i} \partial _{xx} (|\partial _{x} v_{i}^{\varepsilon}|)-4 d_{i} \left ( \frac{f''(v_{i}^{\varepsilon})}{f'(v_{i}^{\varepsilon})} + f'(v_{i}^{\varepsilon}) \right ) \partial _{x}{ ([\partial _{x} v_{i}^{\varepsilon}]^{2})} \partial _{x} v_{i}^{\varepsilon}\\ &\quad{} + [d_{i}^{\frac{1}{3}}|\partial _{x} v_{i}^{\varepsilon}| - \theta (T,R_{0})]^{3}< 0 \end{aligned}$$

Following, the Appendix B of [8], the conclusion follows by comparing $|\partial _{x} v_{i}^{\varepsilon}|$ to $\frac{1}{2\sqrt{t}} + \theta (T,R)$. We conclude that

$$ \max (|\partial _{x} u_{1}^{\varepsilon}|, |\partial _{x} u_{2}^{\varepsilon}|)(x,t) \leq \frac{C(t_{1}, T) e^{C_{\delta}R}}{\sqrt{t_{1}}} \qquad \text{ for } \quad (x,t) \in [0, R] \times [t_{1}, T]. $$

 □

#### Corollary 3.5

*Under the hypotheses* (H1), (H2), (H3) *and* (H4) *we have*

$$ \max (|\partial _{x} u_{1}^{\varepsilon}|, |\partial _{x} u_{2}^{\varepsilon}|)(x,t) \leq C(t_{1}, T) e^{C_{\delta}x} \qquad \textit{ for } \quad (x,t) \in \mathbb{R}^{+} \times [t_{1}, T] $$

(*where* $C(t_{1}, T)$ *is a new arbitrary large constant that depends only on* $t_{1}$ *and* $T$).

### Asymptotic of $u_{i}^{\varepsilon}- u_{j}^{\varepsilon}$

We only prove the following Lemma

#### Lemma 3.6

*Under the hypotheses* (H1), (H2), (H3) *and* (H4), *for any time interval* $[t_{1}, T]$, *there exists* $C(t_{1},T)> 0$ *such that*

$$ -C(t_{1}, T)(x+1) \leq \ln (q_{i}(x, t)) < C(t_{1},T)(x+1).$$

Indeed, it is sufficient to prove this lemma because the proof of the upper bound point 3. of Theorem 2.2 is the same than the proof of Lemma 3.3 by replacing $q_{i}^{+}$ by $q_{i}$ and by using the lower bound provided by Lemma 3.6 instead of the estimate provided by Lemma 3.2. Notice that the proof of the lower bounds follows exactly the same argument than the upper bound except that $P_{d_{i},d_{j}}\left (e^{\frac{(u_{i}-u_{j})(x_{\mu}), \tau _{\mu}}{\varepsilon}}\right )>0$. Therefore, we let the details of the proof for the reader.

#### Proof of Lemma 3.6

We prove this lemma for $i=1$, the proof works the same with $i=2$. We underline that the constant $C(t_{1},T)$ can increase from line to line but does not depend on $x$ or $\varepsilon $.

• **The upper bound.** We start from the definition of $q_{1}$:

3.8 $$ \begin{aligned} \ln ( q_{1}(x,t) ) = &\ln \bigg( [d_{1} -d_{2}](\partial _{x} u_{2}^{\varepsilon}(x,t))^{2} + r_{1}-r_{2} \\ &\qquad + \sqrt{ ( [d_{1} -d_{2}]( \partial _{x} u_{2}^{\varepsilon}(x,t))^{2} + r_{1}-r_{2} )^{2}+ 4 \delta _{1} \delta _{2} } \bigg) \\ & \qquad - \ln ( 2 \delta _{2}). \end{aligned} $$

According to Corollary 3.5, we have that for all $t \in [t_{1}, T]$

$$ \begin{aligned} &[d_{1} -d_{2}](\partial _{x} u_{2}^{\varepsilon}(x,t))^{2} + r_{1}-r_{2} + \sqrt{ ( [d_{1} -d_{2}](\partial _{x} u_{2}^{\varepsilon}(x,t))^{2} + r_{1}-r_{2} )^{2}+ 4\delta _{1} \delta _{2} } \\ \leq \ & (C(t_{1}, T) e^{C_{\delta}x})^{2} \left ( d_{1} +d_{2} + \sqrt{ ( [d_{1} -d_{2}](1 + \frac{r_{1}-r_{2} }{ e^{2C_{\delta}x}})^{2}+ \frac{4\delta _{1} \delta _{2}}{ e^{4C_{\delta}x}} } \right ) + 2 C_{R} \\ \leq \ & C(t_{1},T) (e^{2C_{\delta}x} + 1). \end{aligned} $$

It follows

3.9 $$ \begin{aligned} &\ln \left ([d_{1} -d_{2}](\partial _{x} u_{2}^{\varepsilon}(x,t))^{2} + r_{1}-r_{2} + \sqrt{ ( [d_{1} -d_{2}](\partial _{x} u_{2}^{\varepsilon}(x,t))^{2} + r_{1}-r_{2} )^{2}+ 4\delta _{1} \delta _{2} } \right ) \\ & \leq [2 C_{\delta}x + C(t_{1}, T)]. \end{aligned} $$

Thanks to (H2), we have

3.10 $$ -\ln ( 2 \delta _{2}) \leq C_{\delta}x + C. $$

Inserting (3.9) and (3.10) into (3.8), the conclusion follows for the upper bound.

• **The lower bound.** If $d_{1} \geq d_{2}$, then the result is exactly the one obtained in Lemma 3.2. Therefore, we only consider the case $d_{1}< d_{2}$, in this case we have

$$\begin{aligned} q_{1}(x,t) = & \frac{[d_{1} -d_{2}](\partial _{x} u_{2}^{\varepsilon}(x,t))^{2} + r_{1}-r_{2} + \sqrt{ ( [d_{1} -d_{2}](\partial _{x} u_{2}^{\varepsilon}(x,t))^{2} + r_{1}-r_{2} )^{2}+ 4\delta _{1} \delta _{2} } }{ 2 \delta _{2}} \\ = & \frac{ 2 \delta _{1}}{[d_{2} -d_{1}](\partial _{x} u_{2}^{\varepsilon}(x,t))^{2} + r_{2}-r_{1} + \sqrt{ ( [d_{1} -d_{2}](\partial _{x} u_{2}^{\varepsilon}(x,t))^{2} + r_{1}-r_{2} )^{2}+ 4\delta _{1} \delta _{2} } }. \end{aligned}$$

Next, following similar computations than (3.9) and (3.10) the conclusion follows for the lower bound. □

To finish, we state a Proposition that provides some identity related to $q_{i}$. The proposition follows from straightforward computations that we omit here. However, the following identities will be very useful in the proof of point 1. of Theorem 1.2.

#### Proposition 3.7

*The following identities hold true*:

1. $r_{j} + \delta _{j} q_{i} = r_{H}^{D}(\partial _{x} u_{j})$ *where* $r_{H}^{D}$ *is introduced in* (1.11),
2. $q_{i}^{-1} = \frac{[d_{j} - d_{i}] (\partial _{x} u_{j})^{2} + r_{j} - r_{i} + \sqrt{([d_{j} - d_{i}] (\partial _{x} u_{j})^{2} + r_{j} - r_{i})^{2} + 4\delta _{i} \delta _{j} }}{2 \delta _{i}} $,
3. $r_{i} + \delta _{i} q_{i}^{-1} = r_{H}^{D}(\partial _{x} u_{j})$.

Notice that in the special case $d_{i} = d_{j}$, we recover $q_{i}^{-1} = q_{j}$.

### Time Regularity of $u_{i}^{\varepsilon}$

The local Lipschitz time regularity of $u_{i}^{\varepsilon}$ is an exact transposition of the proof of the time regularity of $u_{\varepsilon}$ in [8] in Sect. 3.5. It is performed with the so-called method of doubling variable and relies mainly on the above bounds about $\partial _{x} u_{i}^{\varepsilon}$. We do not provide this proof and just refer to [8] Sect. 3.5.

## The Hamilton Jacobi Convergence Result

### Proof of Theorem 1.2

We split the proof in several parts:

1. The convergence of $N_{\varepsilon}$,
2. The convergence of $u_{i}^{\varepsilon}$ to $u$ and the control condition,
3. The function $u$ is solution of (1.13),
4. The convergence of $n_{\varepsilon}$.

• **Convergence of** $N_{\varepsilon}$**.** We follow the proofs of Theorem 3.1 of [6] and Theorem 2.4 of [8]. First, we sum the two equations and we integrate with respect to $x$, it follows

$$ N_{\varepsilon}'(t) = \frac{1}{\varepsilon } \int _{0}^{+\infty}(1 \ 1 ) \textbf{ R}(x, N_{\varepsilon}(t)) \textbf{n}_{\varepsilon}(x,t) dx : = J_{\varepsilon}(t) .$$

Notice that for all $t>0$, we have

4.1 $$ J_{\varepsilon}(t) \leq \frac{1}{\varepsilon } C_{R} C_{N}. $$

Next, we differentiate $J_{\varepsilon}$ over time and it follows thanks to (H3)

$$ \begin{aligned} J_{\varepsilon}'(t) & = \frac{J_{\varepsilon}(t) }{\varepsilon } \int _{0}^{+ \infty} (1 \ 1 ) \ \partial _{2} \textbf{R}(x, N_{\varepsilon}) \textbf{n}^{\varepsilon}(x,t)dx + \frac{1}{\varepsilon ^{2}} \int _{0}^{+ \infty}(1 \ 1 )\textbf{ R}(x, N_{\varepsilon}(t))^{2} \textbf{n}^{\varepsilon}(x,t) dx \\ & + \frac{1}{\varepsilon ^{2}} \int _{0}^{+\infty}(1 \ 1 )\textbf{ R}(x, N_{\varepsilon}(t)) \textbf{D} \partial _{xx} \textbf{n}^{\varepsilon}(x,t) dx \\ & = - \frac{N_{\varepsilon}(t) J_{\varepsilon}(t) }{\varepsilon } + \frac{1}{\varepsilon ^{2}}\int _{0}^{+\infty}(1 \ 1 )\textbf{ R}(x, N_{\varepsilon}(t))^{2} \textbf{n}^{\varepsilon}(x,t) dx \\ & + \frac{1}{\varepsilon ^{2}} \int _{0}^{+\infty}(1 \ 1 )\textbf{ R}(x, N_{\varepsilon}(t)) \textbf{D} \partial _{xx} \textbf{n}^{\varepsilon}(x,t) dx \\ & \geq -C_{N} \left (C_{R} + \frac{J_{\varepsilon}}{\varepsilon} \right ) + \frac{1}{\varepsilon ^{2}} \int _{0}^{+\infty}(1 \ 1 ) \textbf{ R}(x, N_{\varepsilon}(t))^{2} \textbf{n}^{\varepsilon}(x,t) dx . \end{aligned} $$

As mentioned in the introduction, a new technical difficulty arises since we deal with a system: it is not clear that the quantity

$$ (1 \ 1 )\textbf{ R}(\cdot , N_{\varepsilon})^{2} \textbf{n}^{\varepsilon}\geq 0. $$

Indeed, using mainly Proposition 3.7 on $q_{i}$, we prove

$$ \frac{1}{\varepsilon ^{2}} \int _{0}^{+\infty}(1 \ 1 )\textbf{ R}(x, N_{\varepsilon}(t))^{2} \textbf{n}^{\varepsilon}(x,t) dx > - C \varepsilon $$

which is enough to conclude to the convergence of $N_{\varepsilon}$ (as we will detail later on). To prove such an inequality, we start from

4.2 $$ \begin{aligned} (1 \ 1 )\textbf{ R}(\cdot , N_{\varepsilon})^{2} \textbf{n}^{\varepsilon}= & n_{1}^{\varepsilon}\left [ (r_{1} - N_{\varepsilon})^{2} + \delta _{1} \delta _{2} + (r_{1} + r_{2} - 2N_{\varepsilon}) \delta _{2} \right ] \\ & \qquad + n_{2}^{\varepsilon}\left [ (r_{2} - N_{\varepsilon})^{2} + \delta _{1} \delta _{2} + (r_{1} + r_{2} - 2N_{\varepsilon}) \delta _{1} \right ] \\ = & (r_{1} + \delta _{2} - N_{\varepsilon}) \left [ (r_{1} - N_{\varepsilon}) n_{1}^{\varepsilon}+ \delta _{1} n_{2}^{\varepsilon}\right ] \\ &\qquad + (r_{2} + \delta _{1} - N_{\varepsilon}) \left [ (r_{2} - N_{\varepsilon}) n_{2}^{\varepsilon}+ \delta _{2} n_{1}^{\varepsilon}\right ]. \end{aligned} $$

Thanks to the point 3. of Theorem 2.2 and Proposition 3.7, we have for $t\geq \varepsilon $

4.3 $$ \begin{aligned} \left [ (r_{2} - N_{\varepsilon}) n_{2}^{\varepsilon}+ \delta _{2} n_{1}^{\varepsilon}\right ] &= n_{2}^{\varepsilon}[ r_{2} - N_{\varepsilon}+ \delta _{2} q_{1} + o(\varepsilon ^{3})] \\ &= n_{2}^{\varepsilon}[r_{H}^{D}(\partial _{x} u_{2}^{\varepsilon}) - N_{\varepsilon}+ o(\varepsilon ^{3})]. \end{aligned} $$

With similar computations, we also have

4.4 $$ \left [ (r_{1} - N_{\varepsilon}) n_{1}^{\varepsilon}+ \delta _{1} n_{2}^{\varepsilon}\right ] = n_{1}^{\varepsilon}\left [ r_{1} + q_{1}^{-1} \delta _{1} - N_{\varepsilon}\right ] = n_{1}^{\varepsilon}\left [ r_{H}^{D}( \partial _{x} u_{2}^{\varepsilon}) - N_{\varepsilon}+ o(\varepsilon ^{3}) \right ]. $$

Inserting (4.3) and (4.4) into (4.2), it follows

$$\begin{aligned} (1 \ 1 )\textbf{ R}(\cdot , N_{\varepsilon})^{2} \textbf{n}^{\varepsilon}& = [r_{H}^{D}(\partial _{x} u_{2}^{\varepsilon}) - N_{\varepsilon}] \left [ (r_{1} + \delta _{2})n_{1}^{\varepsilon}+ (r_{2} + \delta _{1}) n_{2}^{\varepsilon}- N_{\varepsilon}(n_{1}^{\varepsilon}+ n_{2}^{\varepsilon}) \right ] \\ &\quad{} + o(\varepsilon ^{3})(n_{1}^{\varepsilon}+ n_{2}^{\varepsilon}). \end{aligned}$$

Using again the point 3. of Theorem 2.2 and Proposition 3.7, it follows

$$ \begin{aligned} (r_{1} + \delta _{2} - N_{\varepsilon})n_{1}^{\varepsilon}+ (r_{2} + \delta _{1} - N_{\varepsilon}) n_{2}^{\varepsilon}&= (r_{1} + \delta _{2} - N_{\varepsilon}) (q_{1} + o(\varepsilon ^{3}) ) n_{2}^{\varepsilon}+ (r_{2} + \delta _{1} - N_{\varepsilon}) n_{2}^{\varepsilon}\\ & = \left [(r_{1} + \delta _{2} ) q_{1} + r_{2} +\delta _{1} - N_{\varepsilon}(1 +q_{1}) + o(\varepsilon ^{3}) \right ]n_{2}^{\varepsilon}\\ & = n_{2}^{\varepsilon}(1+q_{1}) \left [ \frac{r_{2} + q_{1} \delta _{2} + q_{1} (r_{1} + q_{1}^{-1} \delta _{1}) }{1 + q_{1} } - N_{\varepsilon}\right ] \\ &\quad{} + o(\varepsilon ^{3}) n_{2}^{\varepsilon}\\ & = n_{2}^{\varepsilon}(1 + q_{1}) (r_{H}^{D}(\partial _{x} u_{2}^{\varepsilon}) - N_{\varepsilon}) + o(\varepsilon ^{3}) n_{2}^{\varepsilon}. \end{aligned} $$

We deduce that

$$ \begin{aligned} \frac{1}{\varepsilon ^{2}} \int _{0}^{+\infty}(1 \ 1 )\textbf{ R}(x, N_{\varepsilon})^{2} \textbf{n}^{\varepsilon}(x)dx = & \frac{1}{\varepsilon ^{2}} \int _{0}^{+\infty} (1 + q(x)) [r_{H}^{D}(x, \partial _{x} u_{2}^{\varepsilon}) - N_{\varepsilon}]^{2} n_{2}^{\varepsilon}(x,t) dx \\ &\qquad + o(\varepsilon ) \int _{0}^{+\infty}(n_{1}^{\varepsilon}+ 2 n_{2}^{\varepsilon})(x, t) dx \\ \geq &- 2 C \varepsilon . \end{aligned} $$

We conclude that for all $t > \varepsilon $, we have

$$ J_{\varepsilon}' (t) > -C_{N}\left ( \frac{ J_{\varepsilon}(t)}{\varepsilon} + C_{R}\right ) - 2C \varepsilon .$$

By integrating the above inequality between $t=\varepsilon $ and $t$, we deduce thanks to (4.1)

$$ \begin{aligned} & J_{\varepsilon}(t) > \left [J_{\varepsilon}(\varepsilon ) + \frac{2C \varepsilon ^{2} }{C_{N}} + C_{R} \varepsilon \right ] e^{\frac{-C_{N} t }{\varepsilon}} -\left ( \frac{ 2C\varepsilon ^{2} }{C_{N}} + C_{R} \varepsilon \right ) e^{C_{N}} \\ &\geq \left [\frac{C_{R} C_{N} }{\varepsilon} + \frac{2C \varepsilon ^{2} }{C_{N}} + C_{R} \varepsilon \right ] e^{\frac{-C_{N} t }{\varepsilon}} -\left ( \frac{ 2C\varepsilon ^{2} }{C_{N}} + C_{R} \varepsilon \right ) e^{C_{N}} \\ &>- O_{\varepsilon}(1). \end{aligned} $$

Finally, following the Annex B of [8], we fix $\tau >0$ and it follows for $\varepsilon <\tau $

$$ \int _{\tau}^{T} | N_{\varepsilon}'(s)| ds = \int _{\tau}^{T} N_{\varepsilon}'(s)ds + 2\int _{\tau}^{T} \max ( 0, N_{\varepsilon}'(s)) ds \leq C_{N} - c_{N} + 2(T - \tau ) O_{\varepsilon}(1). $$

We conclude thanks to the compact embedding of $W^{1,1}([\tau ,T])$ into $L^{q}([\tau , T])$. Up to a subsequence, $N_{\varepsilon}$ converges to a function $N$ on every interval of the form $[\tau , T]$ for every $\tau >0$. By a diagonal process, we conclude to the convergence of $N_{\varepsilon}$ on $]0, +\infty [$. Moreover, it is clear that $N$ is non-decreasing.

• **Convergence of** $u_{i}^{\varepsilon}$**.** From the points 1, 2 and 4 of Theorem 2.2, we deduce thanks to the Arzela-Ascoli Theorem that $u_{i}^{\varepsilon}$ converges uniformly on any set of the form $]0, R[ \times ]\tau , T[$ with $R, T$ arbitrary large constants and $\tau $ an arbitrary small constant. We deduce that $u_{i}^{\varepsilon}$ converges uniformly locally on $[0, +\infty [ \times ]0, +\infty [$. Moreover, thanks to the point 3 of Theorem 2.2, we deduce that

$$ \underset{ \varepsilon \to 0}{\lim } \ u_{1}^{\varepsilon}(x,t)= \underset{ \varepsilon \to 0}{\lim } \ u_{2}^{\varepsilon}(x,t)= u(x,t).$$

Next, we claim that $u(x,t) \leq 0$. We prove it by contradiction: assume that there exists a time $t>0$ and $x\in \mathbb{R}^{+}$ such that $u(x,t)>\alpha >0$. We deduce the existence of a sequence $\left ((x_{k}, t_{k}), \varepsilon _{k}\right ) \to \left ((x,t), 0 \right )$ such that $u_{i}^{\varepsilon _{k}} (x_{k}, t_{k})>\frac{\alpha}{2}$. Next, according to the point 2 of Theorem 2.2, there exists a radius $r>0$ such that for all $y \in B(x_{k},r)$, there holds

$$ u_{i}^{\varepsilon _{k}}(y, t_{k}) > \frac{\alpha}{4}.$$

It follows that for $\varepsilon _{k}$ small enough, $N_{\varepsilon _{k}} > C_{N}$ which is in contradiction with the conclusion of Theorem 1.1.

We finally claim that for all $t>0$, we have $\underset{x \in \mathbb{R}_{+} }{\sup}\ u(x,t) = 0$. Assume that the conclusion does not hold true. It follows the existence of a time $t>0$ such that $u(x,t) < -\alpha < 0$. We deduce that for $\varepsilon $ small enough, we have

$$ u_{i}^{\varepsilon}(x, t) \leq \frac{-\alpha}{2} \qquad \forall x > 0.$$

We conclude that for $\varepsilon $ small enough, $N_{\varepsilon}< c_{N}$ which is in contradiction with the conclusion of Theorem 1.1.

• **The function** $u$ **is solution of** (**1.13**)**.** We first prove that $u$ is a super-solution in a viscosity sense of $\partial _{t} u - \mathcal{H}_{D}(\partial _{x} u, N) = 0$. We proceed as it was introduced in the article [8]. Let $(x_{0}, t_{0}) \in \mathbb{R}^{+} \times \mathbb{R}^{+}$ and $\phi $ be a regular test function such that

$$ \min (u - \phi ) = (u - \phi )(x_{0}, t_{0}). $$

Then, we notice that

$$ u(x,t) =\underset{\varepsilon \to 0}{\lim} \ u_{1}^{\varepsilon}(y,s) - \varepsilon \ln \left (\psi _{1}^{\rho _{0}}\right ) = \underset{\varepsilon \to 0}{\lim} \ u_{2}^{\varepsilon}(y,s) - \varepsilon \ln \left (\psi _{2}^{\rho _{0}}\right ) $$

where

$$ \rho _{0} = \partial _{x} \phi (x_{0}, t_{0}) \quad \text{ and } \quad \psi ^{\rho _{0}} = \begin{pmatrix} \psi _{1}^{\rho _{0}} \\ \psi _{2}^{\rho _{0}} \end{pmatrix} = \begin{pmatrix} 1 \\ \frac{ (d_{1} - d_{2}) {\rho _{0}}^{2} + (r_{1} - r_{2}) + \sqrt{(d_{1} - d_{2}) {\rho _{0}}^{2} + (r_{1} - r_{2})^{2} + 4 \delta _{1} \delta _{2} }}{2\delta _{2}} \end{pmatrix} . $$

The function $\psi ^{\rho _{0}}$, introduced in (1.12), is a positive eigenvector of ${\rho _{0}}^{2} \textbf{D} + \textbf{R}$ associated to the eigenvalue $\mathcal{H}_{D}(\partial _{x} \phi (x_{0}, t_{0}), N_{\varepsilon})$. We deduce

4.5 $$ \begin{aligned} &\exists \varepsilon _{k} \underset{ k \to +\infty}{\longrightarrow} 0, \quad \varepsilon _{k}>0, \quad \exists (x_{k}, t_{k}) \in \mathbb{R}^{+} \times \mathbb{R}^{+}\qquad \text{ such that } (x_{k} ,t_{k}) \to (x_{0}, t_{0}) \\ & (u_{i}^{\varepsilon _{k}}- \varepsilon _{k} \ln (\psi _{i}^{\rho _{0}}) -\phi )(x_{k}, t_{k}) = \min \left [(u_{1}^{\varepsilon _{k}}- \phi ) (x_{k}, t_{k}) , ( u_{2}^{\varepsilon _{k}} - \varepsilon _{k} \ln (\psi _{2} ^{ \rho _{0}}) - \phi )(x_{k}, t_{k}) \right ] , \\ &\text{and } \ [u_{i}^{\varepsilon _{k}} - \varepsilon _{k} \ln (\psi _{i} ^{\rho _{0}}) - \phi ](x_{k},t_{k}) = \underset{\mathbb{R}^{+} \times \mathbb{R}^{+} }{\min} [u_{i}^{ \varepsilon _{k}} - \varepsilon _{k} \ln (\psi _{i} ^{\rho _{0}}) - \phi ]. \end{aligned} $$

As we have denoted $\rho _{0} = \partial _{x} \phi (x_{0}, t_{0}) $, we will denote $\rho _{k} = \partial _{x} \phi (x_{k}, t_{k}) $. Notice that $\rho _{k} \to \rho _{0}$. Since it is a minimum point, it follows

$$ \begin{aligned} &\partial _{t} [u_{i}^{\varepsilon _{k}} - \varepsilon _{k} \ln ( \psi _{i} ^{\rho _{0}}) - \phi ](x_{k},t_{k}) = 0, \quad \partial _{x} [u_{i}^{\varepsilon _{k}} - \varepsilon _{k} \ln (\psi _{i} ^{\rho _{0}} ) - \phi ](x_{k},t_{k}) =0 \quad \\ \text{ and } \quad -&\partial _{xx} [u_{i}^{\varepsilon _{k}} - \varepsilon _{k} \ln (\psi _{i} ^{\rho _{0}}) - \phi ](x_{k},t_{k}) \leq 0.\end{aligned} $$

Using the equation (1.9), we deduce that

$$\begin{aligned} 0 &\leq \left ( \partial _{t} \phi + \varepsilon _{k} d_{i} \partial _{xx}( \phi + \varepsilon _{k} \ln (\psi _{i} ^{\rho _{0}} )) - d_{i} [ \partial _{x} \phi + \varepsilon _{k} \partial _{x}\ln (\psi _{i} ^{ \rho _{0}} )]^{2} - r_{i} +N_{\varepsilon}(t) -\delta _{i} e^{ \frac{u_{j}^{\varepsilon _{k}} - u_{i}^{\varepsilon _{k}}}{\varepsilon _{k}}} \right )\\&\quad{}\times (x_{k},t_{k}) . \end{aligned}$$

Moreover, according (4.5), we have

$$ (u_{i}^{\varepsilon _{k}} - u_{j}^{\varepsilon _{k}} )(x_{k}, t_{k} ) \leq \varepsilon _{k} \left [\ln (\psi _{i}^{\rho _{0}}(x_{k}) ) - \ln ( \psi _{j} ^{\rho _{0}}(x_{k}) )\right ].$$

It follows

$$ \begin{aligned} 0 \leq &\left ( \partial _{t} \phi + \varepsilon _{k} d_{i} \partial _{xx}( \phi +\varepsilon _{k} \ln (\psi _{i} ^{\rho _{0}})) - d_{i} ( \partial _{x} \phi + \varepsilon _{k} \partial _{x} \ln (\psi _{i} ^{ \rho _{0}}))^{2} - r_{i}+N_{\varepsilon}(t) - \frac{\delta _{i} \psi _{j} ^{\rho _{0}}}{\psi _{i} ^{\rho _{0}}} \right )\\&\quad{}\times (x_{k},t_{k}) \\ = & \partial _{t} \phi (x_{k}, t_{k}) - \left ( d_{i} (\partial _{x} \phi )^{2} +r_{i} - N_{\varepsilon}+ \frac{\delta _{i} \psi _{j}^{\rho _{k}}}{\psi _{i}^{\rho _{k}}} \right ) (x_{k},t_{k}) \\ +& d_{i}\left ( \varepsilon _{k} \partial _{xx} (\phi +\varepsilon _{k} \ln (\psi _{i} ^{\rho _{0}})) - 2\varepsilon _{k} \partial _{x} \phi \partial _{x} \ln (\psi _{i} ^{\rho _{0}}) - \varepsilon _{k}^{2} ( \partial _{x} \ln (\psi _{i} ^{\rho _{0}}))^{2} \right ) (x_{k}, t_{k}) \\ -& \delta _{i}(x_{k}) \left ( \frac{ \psi _{j}^{\rho _{k}}}{\psi _{i}^{\rho _{k}}} - \frac{ \psi _{j}^{\rho _{0}}}{\psi _{i}^{\rho _{0}}} \right ) \\ & = \partial _{t} \phi (x_{k}, t_{k}) - \left ( d_{i} (\partial _{x} \phi )^{2} + r_{i} - N_{\varepsilon}+ \frac{\delta _{i} \psi _{j}^{\rho _{k}}}{\psi _{i}^{\rho _{k}}} \right ) (x_{k},t_{k}) + o_{\varepsilon _{k}}(1). \end{aligned} $$

Moreover, recalling that $\mathcal{H}_{D}(\partial _{x} \phi (x_{k}, t_{k}), N_{\varepsilon})$ is an eigenvalue of $\partial _{x} \phi ^{2}(x_{k}, t_{k}) \textbf{D} + \textbf{R}$, it follows

$$ \begin{aligned} & (d_{i}[\partial _{x} \phi ]^{2} + r_{i} - N_{\varepsilon _{k}}) \psi _{i}^{\rho _{k}} + \left (\delta _{i} \psi _{j}^{\rho _{k}} \right )(x_{k}, t_{k}) = \left (\mathcal{H}_{D}(\partial _{x} \phi , N_{\varepsilon}) \psi _{i}^{\rho _{k}} \right )(x_{k}, t_{k}) \\ \Rightarrow \quad &\left ( d_{i} (\partial _{x} \phi )^{2} -r_{i} + N_{\varepsilon}- \frac{\delta _{i} \psi _{j}^{\rho _{k}}}{\psi _{i}^{\rho _{k}}} \right )(x_{k}, t_{k}) = \mathcal{H}_{D}(\partial _{x} \phi , N_{\varepsilon}) (x_{k}, t_{k}). \end{aligned} $$

We deduce that

$$ 0 \leq \partial _{t} \phi (x_{k}, t_{k}) - \mathcal{H}_{D}(\partial _{x} \phi , N_{\varepsilon _{k}} )(x_{k} , t_{k}) +o_{\varepsilon _{k}}(1) .$$

Taking the limit $k \to +\infty $, we conclude that $u$ is a super-solution of $\partial _{t} u - \mathcal{H}_{D}(\partial _{x} u , N) = 0$ in a viscosity sense.

It remains to prove the limit conditions: we verify that $u$ satisfies in a viscosity sense $-\partial _{x} u (x=0, t) = 0$. Let $\phi $ be such that $u-\phi $ takes its minimum at $x=0$ and for some positive time $t$. We deduce the existence of $(x_{\varepsilon}, t_{\varepsilon})$ such that

$$ \begin{aligned} &x_{\varepsilon}\to 0, \quad t_{\varepsilon}\to t \text{ as } \varepsilon \to 0, \\ &[u_{i}^{\varepsilon}- \varepsilon \ln (\psi _{i} [\partial _{x} \phi (0, t)]) - \phi ](x_{\varepsilon}, t_{\varepsilon}) \\ &\quad = \min \left ((u_{1}^{ \varepsilon} - \phi ) (x_{\varepsilon}, t_{\varepsilon}), (u_{2}^{ \varepsilon} - \varepsilon \ln (\psi _{2}[\partial _{x} \phi (0, t)])) (x_{\varepsilon}, t_{\varepsilon}) \right ) \\ \text{ and } \quad & [u_{i}^{\varepsilon}- \varepsilon \ln (\psi _{i}[ \partial _{x} \phi (0, t)]) - \phi ](x_{\varepsilon}, t_{\varepsilon}) \\ &\quad = \underset{(x,t) \in \mathbb{R}^{+} \times \mathbb{R}^{+} }{\min } [u_{i}^{ \varepsilon}- \varepsilon \ln (\psi _{i}[\partial _{x} \phi (0, t)]) - \phi ](x,t). \end{aligned} $$

We distinguish two cases:

1. *Case 1:* $x_{\varepsilon}>0$*.* In this case, we conclude exactly as above that
  $$ 0 \leq \partial _{t} \phi (x_{\varepsilon}, t_{\varepsilon}) - \mathcal{H}_{D}(\partial _{x} \phi (x_{\varepsilon}, t_{\varepsilon}), N_{\varepsilon}) +o_{\varepsilon}(1) .$$
2. *Case 2:* $x_{\varepsilon}= 0$*.* In this case, using the fact that $(x_{\varepsilon}, t_{\varepsilon})$ is a minimum point, we deduce that
  $$ -\partial _{x} [u_{i}^{\varepsilon}- \varepsilon \ln (\psi _{i}[ \partial _{x} \phi (0, t)]) - \phi ](0, t_{\varepsilon})\leq 0.$$
  Next, according to the Neumann boundary conditions imposed to $u_{i}^{\varepsilon}$, we deduce that
  $$ \varepsilon \partial _{x}( \ln (\psi _{i} [\partial _{x} \phi (0, t)]) ) (0) \leq -\partial _{x} \phi (0, t_{\varepsilon}) . $$

Passing to the superior limit $\varepsilon \to 0$

$$ 0 \leq \max \left ( -\partial _{x} \phi (0, t) , \phi (0, t) - \mathcal{H}_{D}(\partial _{x} \phi (0, t) , N_{\varepsilon}(t))\right )$$

which corresponds to the boundary conditions in a viscosity sense.

The proof that $u$ is a sub-solution of (1.13) follows from the same arguments.

• **Convergence of** $n_{i}^{\varepsilon}$ **in the sense of measures.** The proof that $n_{i}^{\varepsilon}$ converges to a measure follows from the convergence of $u_{i}^{\varepsilon}$ towards $u$. Indeed, fix times $0 < t_{1}< T$; then, according to point 1. of Theorem 2.2, there exists $R_{T}>0$ such that for any $x>R_{T}$, $t\in (t_{1}, T)$ and $\varepsilon $ small enough, we have

$$ u_{i}^{\varepsilon}(x,t) \leq C(1 - x) \quad \text{ and } \quad \int _{ \left \lbrace x > R_{T} \right \rbrace } n_{1}^{\varepsilon}(x,t) + n^{\varepsilon}_{2}(x,t) dx \leq \frac{c_{N}}{2}. $$

Hence, we deduce that $n_{i}^{\varepsilon}\to 0$ on $\left \lbrace x > R_{T} \right \rbrace $. It follows

$$ \begin{aligned} \frac{ c_{N} }{2\left (1 + \underset{ x \in [0, R_{T}]}{\min} q_{1}(x)e^{\frac{\varepsilon ^{3}}{t_{1}}} \right ) }& \leq \frac{ 1 }{\left (1 + \underset{ x \in [0, R_{T}]}{\min} q_{1}(x)e^{\frac{\varepsilon ^{3}}{t_{1}}} \right ) } \int _{\left \lbrace x < R_{T} \right \rbrace } (n_{1}^{\varepsilon}(x,t) + n_{2}^{\varepsilon}(x,t)) dx \\ &\leq \frac{ 1 }{\left (1 + \underset{ x \in [0, R_{T}]}{\min} q_{1}(x)e^{\frac{\varepsilon ^{3}}{t_{1}}}\right ) } \int _{\left \lbrace x < R_{T} \right \rbrace } n_{1}^{\varepsilon}(x, t) (1 + q_{1}(x) e^{\frac{\varepsilon ^{3}}{t}}) dx \\ &\leq \int _{\left \lbrace x < R_{T} \right \rbrace } n_{1}^{\varepsilon}(x, t) dx \leq C_{N}. \end{aligned} $$

Since the same type of inequalities is valid for $n_{2}^{\varepsilon}$, we deduce that up to an extraction we have $(n_{1}^{\varepsilon}, n_{2}^{\varepsilon}) \to (n_{1}, n_{2})$, where $n_{1}$ and $n_{2}$ are two non-trivial measures. Next, we prove that

$$ \mathrm{supp} \ n_{i}(\cdot , t) \subset \left \lbrace u(\cdot , t) = 0 \right \rbrace . $$

Let a time $t>0$ and $\phi $ be a positive regular compactly supported test function, such that

$$ \mathrm{supp} \ \phi \subset \left \lbrace u(\cdot , t) = 0 \right \rbrace ^{c}.$$

We deduce that there exists $a>0$ such that

$$ \underset{ x \in \mathrm{supp} \ \phi}{\max} u( x , t ) < -a. $$

Hence, for $\varepsilon $ small enough, we have $\underset{ x \in \mathrm{supp} \ \phi}{\max} u_{i}^{\varepsilon}( x , t ) < -\frac{a}{2}$. The conclusion follows the following computation:

$$ \int _{\mathbb{R}^{+}} \phi (x) n (x, t) dx = \underset{\varepsilon \to 0}{\lim} \int _{\mathbb{R}^{+}} \phi (x) n_{i}^{\varepsilon}(x, t) dx \leq \underset{\varepsilon \to 0}{\lim} \int _{ \mathbb{R}^{+}} \phi (x) e^{-\frac{a}{2\varepsilon}} dx = 0. $$

 □

## Optimal Timing and Heterogeneity in the Adaptation to DNA Damage

### A General Non-local System Modelling Adaptation to DNA Damage

We now describe with more mathematical details the model used in [44] and we build a more complex model involving coupled non-local partial differential equations.

The initial population is composed of a quantity $D(0)$ of damaged cells. These cells are assumed to be the only survivors of an event that damaged their DNA and killed part of them. They first try to repair their DNA and succeed at a time-dependent rate that we assume to be Gaussian:

5.1 $$ \alpha (t) = \alpha _{m} \mathrm{e}^{- \frac{(t-\mu _{a})^{2}}{2\sigma}}, $$

where $t$ is the time elapsed since the damage, $\alpha _{m}>0$ is the maximal value of the rate, $\sigma >0$ the variance and $\mu _{a}>0$ the mean. We assume that the cells adapt with a time-dependent rate of logistic type:

5.2 $$ \beta ( x, p , t) = \dfrac{\beta _{m}}{1+ \mathrm{e}^{-p(t- x )}}. $$

This rate involves the parameters $x$ of the adaptation timing that represent the center of the curve and $p$ which represents the heterogeneity. We also fix the hyper-parameter $\beta _{m}>0$ for the maximal value of this rate.

Adapted cells and their progeny have access to other repair mechanisms at later stages of the cell cycle and we assume that they manage to repair their DNA damage at a constant low rate $\delta >0$. The values $\gamma _{d}>0$ and $\gamma _{a}>0$ are the death rates of damaged and adapted cells respectively; the death rate of healthy cells is assumed to be 0 for the sake of clearness.

Currently, there are not enough data to justify a precise choice of the fixed parameters $\alpha _{m},\beta _{m}, \sigma $ and $\mu _{a}$, but insights from experimental biologists suggest orders of magnitudes and approximate values. In this work, we stick to the choices of [44], which come from discussions with experimental biologists:

$$ \gamma _{d}=0.1,\quad \gamma _{a} = 0.35,\quad \alpha _{m}=\beta _{m}=0.5 ,\quad \sigma =0.5, \quad \mu _{a}=1.$$

The choice of 0 for the death rate of healthy cells is not important since what matters in the model is the relative values of the different death rates. The real values of these death rates in the wild is very hard to determine and depends on many varying factors like predators and environmental conditions.

With these choices of parameters and functions, the model of [44] is defined by the following system of ordinary differential equations:

$$\begin{aligned} \dfrac{d }{d t} \left ( \textstyle\begin{array}{c} D(t) \\ A(t) \\ R(t) \end{array}\displaystyle \right ) & = \left ( \textstyle\begin{array}{c@{\quad}c@{\quad}c} -\gamma _{d} - \beta (x,p,t) -\alpha (t) & 0 & 0 \\ \beta (x,p,t) & -\gamma _{a} - \delta & 0 \\ \alpha (t) & \delta & 0 \end{array}\displaystyle \right )\left ( \textstyle\begin{array}{c} D(t) \\ A(t) \\ R(t) \end{array}\displaystyle \right ) \\&\quad{}+ \left ( \textstyle\begin{array}{c} 0 \\ A(t)(1-\frac{N(t)}{N_{max}}) \\ R(t)(1-\frac{N(t)}{N_{max}}) \end{array}\displaystyle \right ), \end{aligned}$$

where, at time $t$, $D(t)$ is the quantity of damaged cells, $A(t)$ the quantity of adapted cells and $R(t)$ the quantity of healthy cells whose DNA is repaired. We denote

$$ N(t) = A(t)+R(t)+D(t)$$

the total population at time $t$. This system converges to a situation where the healthy cells fill entirely the carrying capacity $N_{max}>0$: $(D(t), A(t), R(t)) \to (0,0,N_{max})$ when $t\to +\infty $.

Depending on the value $x\in \mathbb{R}_{+}$ of the timing of adaptation (or the value $p\in \mathbb{R}_{+}$ when we study heterogeneity), the population will take a certain time $T_{S}(x)$ (respectively $T_{S}(p)$) to reach some arbitrary level near the carrying capacity $N_{max}$ of the system. The authors of [44] observe that there exists for most values of $p$ an optimal value $x^{*}(p)$ which minimises $T_{S}$, thus allowing the population to grow back to a healthy size as fast as possible after an external event has damaged the DNA of all cells. The authors also investigate the dependency of $T_{S}$ with respect to the heterogeneity parameter $p$ when $x$ is fixed. As we explained in the introduction, for realistic values of $x$, $\mathrm{argmin}_{p\geqslant 0} T_{S}(p) = +\infty $, which is in contrast with experimental data that indicate that adaptation is quite heterogeneous in time. The authors of [44] then improve their model, assuming that the source of the initial damage (heat, X-rays, chemicals in the medium,…) is still present for some time and prevents repair. This is modelled by a random variable determining when repair becomes possible after the damage. With this component and minimising the expectancy $\mathbb{E}[T_{S}(p)]$ with respect to the law of the environmental random variable, they find an optimal value

$$ p^{*} = \mathrm{argmin}_{p\geqslant 0}\ \mathbb{E}[T_{S}(p)] $$

for the heterogeneity parameter $p$.

Here we go further into investigating the selection of optimal adaptation timing $x^{*}$ and heterogeneity $p^{*}$. Instead of studying for each value $x$ of the genetic trait the evolution after a damaging event in the isogenic population, we consider a population of cells with varying genetic trait competing for the same resources in an environment.

Consider for example the trait $x$ representing the timing of adaptation. Let $n( x ,t)$ represent at time $t$ the density of healthy cells with genetic trait $x $; let $d( x , s, t)$ represent at time $t$ the density of cells with genetic trait $x $ whose DNA is damaged since a time $s$; let $a( x ,t)$ represent the density of adapted cells at time $t$ with genetic trait $x $. We now assume that some cells are damaged at rate $\mathcal {D}(t)$. We model genetic diffusion in the variable of interest ($x$ or $p$) for both healthy and adapted cells with a Laplacian with diffusion coefficients $d_{1}>0$ (healthy cells) and $d_{2}>0$ (adapted cells). The adapted cells having higher genomic instability, it can be interesting to consider the case $d_{2}>d_{1}$. Last, we directly introduce the scaling parameter $\varepsilon >0$ in the spirit of the model (1.2). As mentioned in the introduction, the parameter $\varepsilon $ accelerates time and makes the mutations rare. In this regime, we expect that only the traits with a positive or null growth rate will be selected. Therefore, we expect that the set of solutions converges to moving Dirac masses.

The repair rate can now take into account both an absolute time $t$ part and a “time $s$ elapsed since the damage occurred” part:

5.3 $$ \alpha (s,t) = \bar{\alpha}(t) \mathrm{e}^{- \frac{(s-\mu _{a})^{2}}{2\sigma}}, $$

where the function $\bar{\alpha}:\mathbb{R}_{+}\to [0,\alpha _{m}]$ allows us to take into account environmental events that prevent cells from repairing their DNA damage, like X-rays, anomalous heat or toxic chemicals. The adaptation rate can depend on $x$ or $p$ depending on what we investigate, which we will denote for clarity

**Fig. 1:**
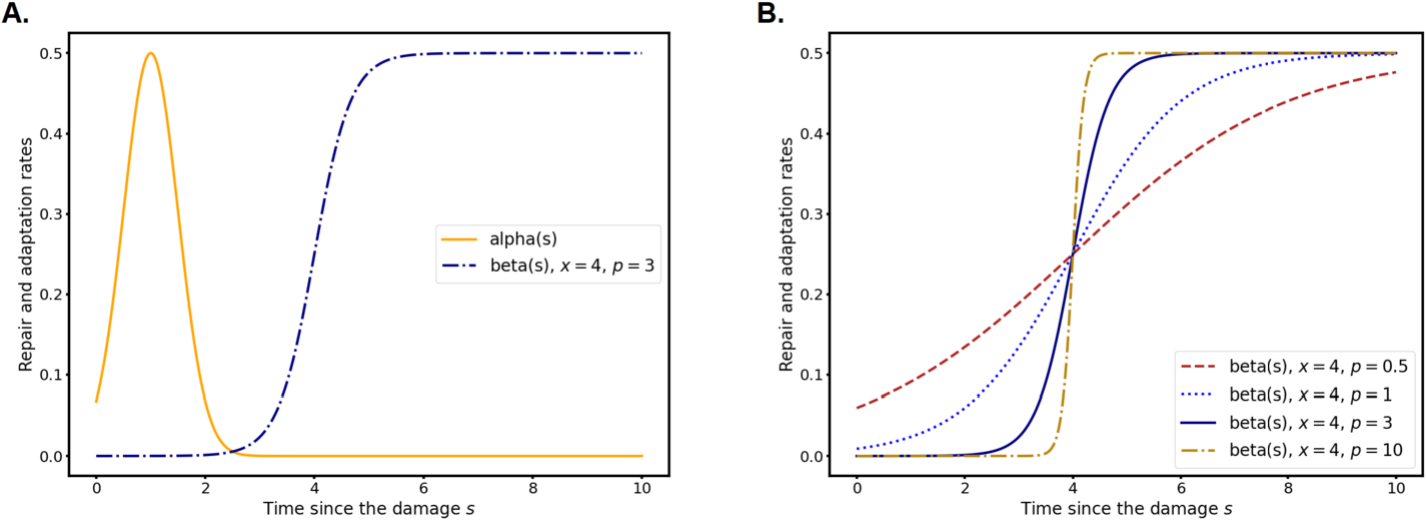
(A) Plot of the rates $\alpha (s,t)$ with $\bar{\alpha}(t) = 0.5$ and $\beta (x,p,s)$ with $x=4$ and $p=3$ in terms of the time $s$ since the damage. (B) Plot of the adaptation rate $\beta (x,p,s)$ in terms of the time $s$ since the damage for $x=4$ and $p=0.5,1,3,10$

5.4 $$ \beta ( x, s) = \dfrac{\beta _{m}}{1+ \mathrm{e}^{-p(s- x )}}, \qquad or \qquad \beta ( p , s) = \dfrac{\beta _{m}}{1+ \mathrm{e}^{-p(s- x )}}, $$

to indicate if cells vary along genetic trait $x$ or $p$ in the model. In Fig. 1 A, we present the rates $\alpha $ and $\beta $ in terms of the time $s$ elapsed since the damage of a cell. In Fig. 1 B, we plot on the same graph the adaptation rate for different values of the heterogeneity parameter $p$ to help visualise what kind of effect varying it has.

Our non-local PDE model writes:

5.5 $$ \varepsilon \dfrac{\partial n}{\partial t}( x ,t) - \varepsilon ^{2}d_{1} \dfrac{\partial ^{2} n}{{\partial x }^{2}} ( x ,t) = n( x ,t)\big( 1 - \mathcal {D}(t) - N(t) \big) +\delta a( x ,t) + \int _{0}^{+\infty} \alpha (s,t)d( x ,s,t) \mathrm{d}s, $$

5.6 $$ \varepsilon \dfrac{\partial d}{\partial t}( x , s, t) + \dfrac{\partial d}{\partial s}( x , s, t) + \big(\gamma _{d} + \alpha (s,t) + \beta ( x ,s) \big)d( x ,s,t) = 0, $$

5.7 $$ \varepsilon \dfrac{\partial a}{\partial t}( x ,t) - \varepsilon ^{2}d_{2} \dfrac{\partial ^{2} a}{{\partial x }^{2}}( x ,t)= a( x ,t)(1-\gamma _{a} - \delta - N(t) ) + \int _{0}^{+\infty}\beta ( x ,s) d( x ,s,t) \mathrm{d}s, $$

5.8 $$ N(t) = \int _{0}^{+\infty} \left ( n( x ,t) + \int _{0}^{+\infty} d( x ,s,t)\mathrm{d}s + a( x ,t)\right ) \mathrm{d}x , $$

with the following initial and boundary conditions

5.9 $$ \left \{ \textstyle\begin{array}{l} \displaystyle \dfrac{\partial n}{\partial x}(0,t)= \dfrac{\partial a}{\partial x}(0,t) = 0,\qquad t\in \mathbb{R}_{+}, \\ \displaystyle d( x ,0,t) = \mathcal {D}(t) n( x ,t),\qquad x ,t\in \mathbb{R}_{+}, \\ \displaystyle n( x ,0)=n^{0}( x ), \quad d( x ,s,0)= d^{0}( x ,s), \quad a( x ,0) = a^{0}( x ), \qquad x \in \mathbb{R}_{+}, \end{array}\displaystyle \right . $$

and where the constants $d_{1}, d_{2}, \delta , \gamma _{d}, \gamma _{a} \in \mathbb{R}_{+}^{*}$ are the same as in the previous model.

Let us comment further on this system in order to justify the different choices we made in the crafting of equations (5.5)–(5.6)–(5.7)–(5.8).

- *Diffusion part:* We assume that mutations can occur when cells reproduce and we choose to model these mutations with a Laplacian in the variable under investigation ($x$ or $p$). Let us recall that other approaches have been studied in the literature, like integral operator with a mutation kernel [8, 40]. Our main assumption in this work, which comes from insights from the experimental community ([20]), is that the value of the parameters $x$ and $p$ can be affected by random mutations, leading to colonies where cells present different behaviours when faced with damage on their DNA.
  In equation (5.5), mutations occur in the healthy population with a rate driven by the diffusion coefficient $d_{1}>0$ and the scaling parameter $\varepsilon >0$; when the later is small, we consider a regime of rare mutations over a long timescale. Although they have chromosomic instability, damaged cells do not have genetic diffusion because, since they are blocked in the cell cycle, they don’t reproduce at all until the damage it either sorted out or ignored. When they adapt, cell division resumes and we model mutations in the adapted population with a Laplacian and a diffusion coefficient $d_{2}>0$. It is expected that $d_{2}>d_{1}$ because of the genetic instability ([20]), but there are currently no experimental data to rigorously support this claim. Estimating mutation rates for organisms is still a widely open question in experimental biology and most systematic review on the topic concern bacteria (*e.g.* [46]).
  Let us also clarify a point about diffusion when we consider $p$ as a variable. The times at which individual cells adapt is then random for two different reasons: first the value of $p$ (the smaller $p$, the more random are adaptation events), then the diversity of the values of $p$ in the population. The effect we want to investigate is the first one, in order to determine which value of $p$ leads to the more fitness in the population, which is why we assume that mutations are rare ($\varepsilon \ll 1$), leading to less heterogeneity in adaptation events due to the diversity of values of $p$ and more heterogeneity due to the value of $p$ which is selected by evolution over long timescales.
- *Reaction part:* Cells are damaged at a rate $\mathcal {D}(t)$ and thus removed from the healthy population $n(x,t)$. These damaged cells enter the damaged population $d$ at time since damage $s=0$ through the boundary condition $d(x,0,t)=\mathcal {D}(t) n(x,t)$. The damaged cells population evolves according to the age-structured hyperbolic equation (5.6). Time since the damage increases at speed $\frac{1}{\varepsilon}$, damaged cells die at rate $\frac{1}{\varepsilon}\gamma _{d}$. The cells damaged at time $t$ since a time $s$ repair their DNA at a rate $\frac{1}{\varepsilon}\alpha (s,t)$. At any time $t$, all cells repaired are put back into the healthy population through the term
  $$ \frac{1}{\varepsilon}\int _{0}^{+\infty} \alpha (s,t)d( x ,s,t) \mathrm{d}s.$$
  In the same fashion, damaged cells adapt at a rate $\frac{1}{\varepsilon}\beta (x,s)$ and at any time $t$ all cells that adapt are put in the adapted population $a(x,t)$ through the term
  $$ \frac{1}{\varepsilon}\int _{0}^{+\infty}\beta ( x ,s) d( x ,s,t) \mathrm{d}s.$$
  Last, all cells compete for resources and are subject to the control of the total population $N(t)$ defined by (5.8). These integrals and the equation (5.6) for damaged cells could be posed on the interval $[0,\frac{t}{\varepsilon}]$ in the $s$ direction since the hyperbolic nature of this equation implies a finite propagation speed. However, it would prevent the use of an arbitrary initial condition $d(x,s,0)$ in the system and other age-structured equations in the literature are posed with $s\in [0,+\infty )$; for these reasons, we pose the equation on an infinite domain for $s$.

### Simplification into a Two Populations System

This system of non-local partial differential equations is hard to tackle numerically for multiple reasons. First, the hyperbolic equation with coupled boundary condition describing damaged cells $d(x,s,t)$ must be solved on a two dimensional domain which may be large in the $s$ direction because when $x$ is not small adaptation events can happen later on. This implies that the more the domain is large in $x$, the more it must be large in $s$. A potential way to circumvent this issue would be a non-square domain, but then it raises the problem of preserving the hyperbolic structure of the equation. More generally, the system possesses conservation properties between $n$, $d$ and $a$ that a robust numerical scheme would have to preserve. Then, every iteration of the system involves the computation of a lot of integrals in the $s$ direction: in the equations for $n$ and $a$ and in the computation of the total mass $N(t)$. Last, when the scaling parameter $\varepsilon $ becomes small, there are no guarantees of numerical stability for this intricate non-local parabolic-hyperbolic system; without proper tuning of the numerical scheme, the time step would have to be small. Numerical study of this model is still possible with a carefully crafted numerical scheme and enough computational power, but this is outside the scope of our paper.

Hence, we simplify the dynamics of the damaged cells by making for $\varepsilon $ small enough the quasi-static approximation

$$ \partial _{s} d(x,s,t) +(\gamma _{d}+ \alpha (s,t) +\beta (x,s)) d(x,s,t) =0. $$

Then, we can compute the quantity of damaged cells explicitly:

$$ d(x,s,t) = \mathcal {D}(t) n(x,t) e^{- \gamma _{d} s - \int _{0}^{s} \alpha (z,t)dz - \int _{0}^{s} \beta (x,z)dz}. $$

We also make the simplifying assumption that the damage rate is constant, *i.e.* $\mathcal {D}(t) = D > 0$, and the new total mass is given by

$$ N_{\varepsilon}(t) = \int _{0}^{+\infty} (n_{\varepsilon}(x,t)+a_{\varepsilon}(x,t)) dx.$$

The damaged cells are absent of this total mass because the quasi-static approximation is tantamount to say that on longer timescales damaged cells are instantaneously distributed between the three categories they go to: healthy, adapted and dead. Since they are instantly redistributed, they don’t need to participate in the carrying capacity in this simpler model.

Hence, we come to the simplified model

5.10 $$ \left \{ \textstyle\begin{array}{rcl} \varepsilon \partial _{t} n_{\varepsilon}(x,t) - \varepsilon ^{2} d_{1} \Delta n_{\varepsilon}(x,t) & = &\displaystyle n_{\varepsilon}(x,t) (1-D-N_{\varepsilon}(t)) + \delta a(x,t) \\ &&\displaystyle \qquad +Dn_{\varepsilon}(x,t)\int _{0}^{\infty}\alpha (s,t) e^{- \gamma _{d} s - \int _{0}^{s} \alpha (z,t)dz - \int _{0}^{s} \beta (x,z)dz}ds, \\ \varepsilon \partial _{t} a_{\varepsilon}(x,t) - \varepsilon ^{2} d_{2} \Delta a_{\varepsilon}(x,t) & = &\displaystyle a_{\varepsilon}(x,t) (1- \gamma _{a}- \delta -N_{\varepsilon}(t)) \\ &&\displaystyle \qquad +Dn_{\varepsilon}(x,t)\int _{0}^{\infty}\beta (x,s) e^{- \gamma _{d} s - \int _{0}^{s} \alpha (z,t)dz - \int _{0}^{s} \beta (x,z)dz}ds, \\ N_{\varepsilon}(t) & = &\displaystyle \int _{0}^{+\infty} (n_{\varepsilon}(x,t)+a_{\varepsilon}(x,t)) dx, \end{array}\displaystyle \right . $$

with the following initial and boundary conditions

5.11 $$ \left \{ \textstyle\begin{array}{l} \displaystyle \dfrac{\partial n_{\varepsilon}}{\partial x}(0,t)= \dfrac{\partial a_{\varepsilon}}{\partial x}(0,t) = 0,\qquad t\in \mathbb{R}_{+}, \\ \displaystyle n_{\varepsilon}( x ,0)=n^{0}( x ), \quad a_{\varepsilon}( x ,0) = a^{0}( x ), \qquad x \in \mathbb{R}_{+}. \end{array}\displaystyle \right . $$

If we choose $\bar{\alpha}(t) = \alpha _{m}$, *i.e.* $\alpha (s,t) = \alpha (s)$, and if we denote

$$\begin{array}{c} r_{1}(x)=1-D+D\int_{0}^{\infty }\alpha (s)e^{-\gamma_{d}s-\int_{0}^{s}\alpha (z)dz-\int_{0}^{s}\beta (x,z)dz}ds,\;r_{2}(x)=1-\gamma_{a}-\delta ,\\ \delta_{1}(x)=\delta \;\text{ and }\;\delta_{2}(x)=D\int_{0}^{\infty }\beta (s)e^{-\gamma_{d}s-\int_{0}^{s}\alpha (z)dz-\int_{0}^{s}\beta (x,z)dz}ds, \end{array}$$

the system (5.10) is of the form (1.5).

If we assume that $D < 1$ and $\gamma _{a} < 1$, then the functions $r_{1}, r_{2}, \delta _{1}, \delta _{2}$ defined above satisfy assumptions (H2) and (H3). Most of the conditions can be readily checked and we postpone the remaining technicalities to the Appendix B. For (H2) the only difficult part is to check that $e^{C_{\delta}x}\delta _{2}(x) \underset{x \to +\infty}{\longrightarrow} +\infty $, which is granted thanks to Lemma B.1. For (H3), thanks to Lemma B.2 and using the fact that

$$ \int _{0}^{\infty}\alpha (s) e^{- \gamma _{d} s - \int _{0}^{s} \alpha (z)dz - \int _{0}^{s} \beta (x,z)dz}ds \leqslant 1, $$

we can choose

$$ c_{N} = \min (1-\delta _{a},1-D)\qquad \mathrm{and} \qquad C_{N} = 2+D.$$

Therefore, we can apply Theorem 1.2 and Theorem 2.2. In particular, if $d_{1} = d_{2}$, then for all $\varepsilon \in \mathbb{R}_{+}^{*}$,

$$ \dfrac{n_{\varepsilon}(x,t)}{a_{\varepsilon}(x,t)} \underset{t\to +\infty}{\longrightarrow} q(x) = \frac{ r_{1}(x) - r_{2}(x) + \sqrt{ (r_{1}(x) - r_{2}(x))^{2} + 4 \delta _{1}(x) \delta _{2}(x)}}{2 \delta _{2}(x)}. $$

Moreover, recall that in the case $d_{1}=d_{2}=1$ the Hamiltonian defined in (1.10) can be decomposed into

$$ \mathcal {H}(\rho ,N) = \rho ^{2} + r_{H}(x) - N, $$

with

$$ r_{H}(x) = \frac{1}{2}\left (r_{1} + r_{2} + \sqrt{(r_{1}-r_{2})^{2} + 4 \delta _{1} \delta _{2} }\right ), $$

the *Hamiltonian fitness*. This function also describes the stationary states as explained in Sect. 2.2. We can compute numerically this function $r_{H}$ to gain insights about the behaviour of the system in the limits $\varepsilon \to 0$ or $t\to +\infty $.

**Fig. 2:**
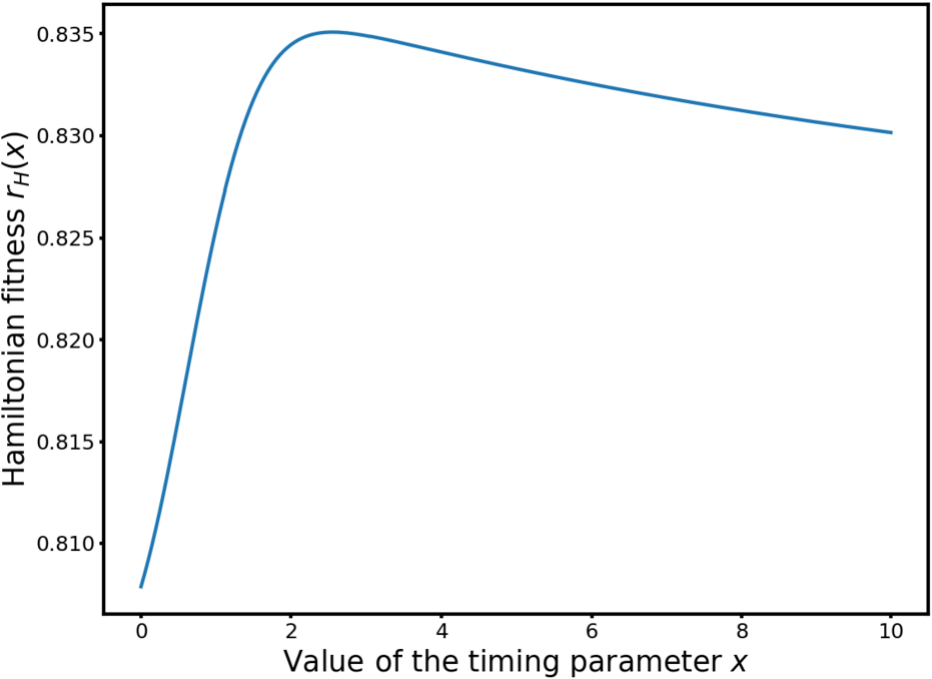
Hamiltonian fitness $r_{H}(x)$ of the system for $p=3$

When the variable of interest is the mean time of adaptation $x$, with fixed $p=\bar{p}$, $r_{H}(x)$ has a unique global maximum as can be seen on Fig. 2. The numerical results in the next section indicate that when $\varepsilon $ goes to 0, the solutions concentrate on a Dirac mass moving towards the maximum point. Hence, this model strengthens the hypothesis of [44] that an optimal timing $x^{*}$ for adaptation tend to be favored by natural selection over long timescales. Here, the optimal time is expressed as

$$ x^{*} = \underset{x\in \mathbb{R}_{+}}{\mathrm{argmax}} \ r_{H}(x). $$

Let us mention that $r_{H}(x)$ should also drive the profile of the stationary state for fixed $\varepsilon $, since, as mentioned above, the formal equation for $w(x) = n_{1,\infty}(x)+n_{2,\infty}(x)$ is

$$ \varepsilon ^{2} \Delta w(x) - r_{H}(x)w(x) = 0. $$

**Fig. 3:**
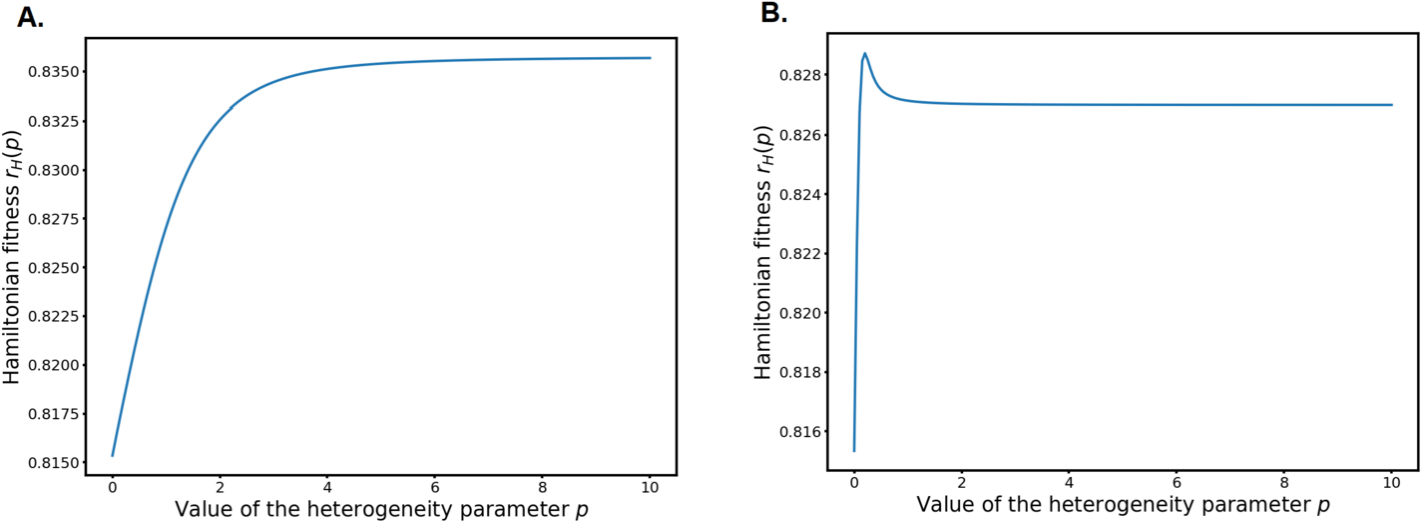
Hamiltonian fitness $r_{H}(p)$ of the system for (A.) $\bar{x}=2$ and (B.) $\bar{x}=20$

If we fix an adaptation timing $x = \bar{x}$ and we take as a variable the adaptation heterogeneity parameter $p$, we can compute another equivalent fitness $r_{H}(p)$ which is displayed in Fig. 3.

As can be seen in Fig. 3A, for “reasonable” values of $\bar{x}$ the function $r_{H}:p\mapsto r_{H}(p)$ is increasing on $\mathbb{R}_{+}$. As we can observe in the numerical simulations in the following section, when $\varepsilon \to 0$ the solutions concentrate on a Dirac mass that moves towards $+\infty $. This is in accordance with the findings of [44] in the simpler ODE model: when the environment is predictable, the optimal strategy for the cells is to minimise the variance around any “good enough” adaptation timing, which amount to taking the largest possible value for $p$.

If the mean adaptation time $\bar{x}$ is large enough, for example $\bar{x}=20$, it can be seen in Fig. 3B that $r_{H}(p)$ has a unique global maximum. Since adaptation is really late, a smaller value $p^{*}$ (i.e. a larger variance for the adaptation) is selected to compensate.

However, as we said in the beginning of this section, in real life experiments the cells adapt with a variable timing. In [44], this fact was explained as a bet-hedging mechanisms in an unpredictable environment. When the optimisation procedure in the variable $p$ has to take into account a random variable in the repair function $\alpha $, a particular value $p^{*}$ is selected. Here we use the absolute-time part $\bar{\alpha}(t)$ in the repair function $\alpha (s,t)$ to model the changing environment. In the next section, we also make numerical experiments to explore what happens to the solution with a time-periodic $\bar{\alpha}$ function.

## Numerical Simulations

In this section, we investigate numerically the behaviour of the system (5.10) with the parameters and functions described in Sect. 5.2.

We use a standard Cranck-Nicolson scheme for the Laplacians and the reaction terms are treated explicitly. This rather simple scheme appears to be very robust even for small $\varepsilon $ values, as long as the time step $dt$ is of the same order of magnitude than $\varepsilon $. The numerical domain $[0,X_{\mathrm{max}}]$ is chosen such that increasing it further has no effect whatsoever on the simulation, which is most of the time $X_{\mathrm{max}} = 12$. The smaller the scaling parameter $\varepsilon $ is, the smaller the numerical domain can be. At both ends of the numerical domain, we use a homogeneous Neumann boundary condition. This method is valid also because we use fast decreasing initial conditions.

Note that the recent preprint [13] proposes a new asymptotic-preserving numerical scheme for this type of equations, along with theoretical guarantees. It could lead to further numerical research in this two-population framework.

The Python code we used to produce the numerical simulations is available at https://github.com/pierreabelroux/Leculier_Roux_2021. The figures can be obtained by running the code.

**Fig. 4:**
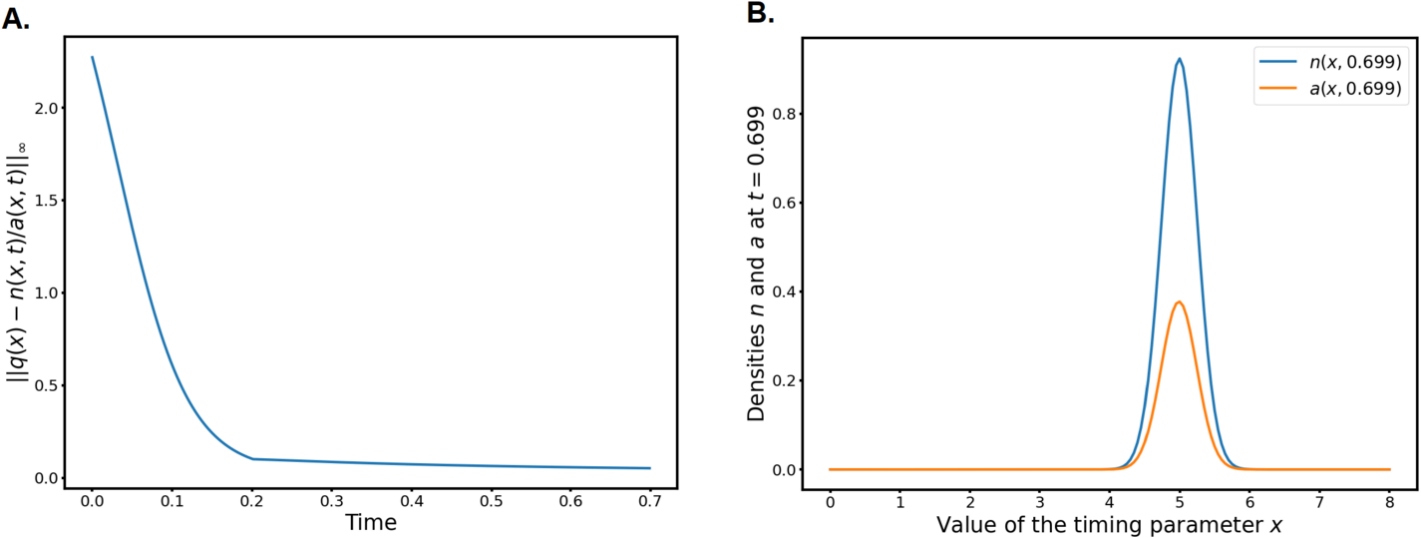
(A.) Distance between $\tfrac{n}{a}$ and $q$ in $L^{\infty}$ norm. The norm is computed over the finite numerical domain $[0,10]$ (B.) Plot of $n(\cdot ,t)$ and $a(\cdot ,t)$ for $t=0.699$. The diffusion parameters are $d_{1}=d_{2}=1$

As can be seen on Fig. 4, the convergence of the quantity $\left \lVert q(\cdot ) - \tfrac{n(\cdot ,t)}{a(\cdot ,t)}\right \rVert $ is very fast and the shapes of $n$ and $a$ are similar right after a short transitory period. In this figure and the following ones we take

$$ n^{0} = a^{0} = \frac{1}{5} e^{-10(x-5)^{2}},$$

to avoid visual scaling problems with $\tfrac{n}{a}$ (this quotient can be very large in the first milliseconds for Gaussians with distant means) but it does not affect the speed of convergence towards $q(x)$ which is consistent across all types of initial data.

Consequently, we will only plot $n(\cdot ,t)$ in the following numerical experiments for the sake of clarity.

### Evolution Along the Parameter $x$ for Fixed $p$

**Fig. 5:**
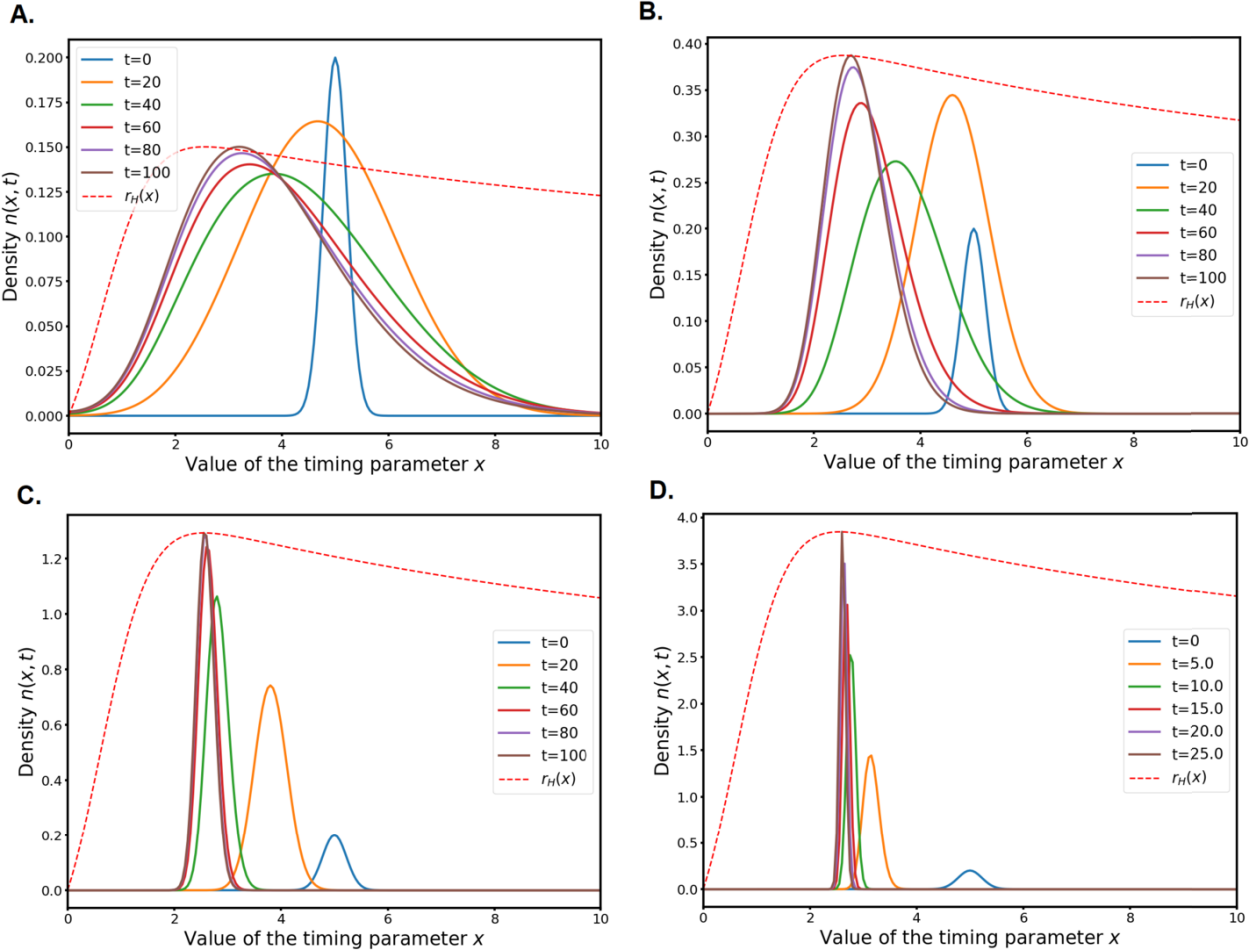
Evolution in time of $n(x,t)$ from the same initial data for $p=3$ and different values of $\varepsilon $. The dashed line is the Hamiltonian fitness $r_{H}(x)$ which is re-scaled for the sake of readability. (A.) $\varepsilon = 0.05$ (B.) $\varepsilon = 0.01$ (C.) $\varepsilon = 0.001$ (D.) $\varepsilon = 0.0001$

For the fixed values $p=3$ and $d_{1}=d_{2}=1$, we simulate the system (5.10) for different values of $\varepsilon $ (see Fig. 5). As predicted by our theoretical results, when $\varepsilon $ tends to 0 the solution behaves like a Dirac mass moving towards the maximum point of the Hamiltonian fitness $r_{H}(x)$. This corresponds to the selection of an optimal mean value for the timing of the adaptation process. In laboratory experiments on budding yeasts ([20, 25, 28]), researchers use mutant populations of cells that are incapable of repairing heat-induced or radiation induced damage. They observe that despite repair being impossible, adaptation occur at a specific timing. The theoretical and numerical results of our model strengthen the hypothesis that this specific average timing is driven by natural selection. Note that this natural selection mechanism is not something that experiments can test currently, which makes model aided hypotheses quite useful.

Yet, taking $d_{1}=d_{2}$ is not realistic from a biological point of view in this context. It is observed in experiments that adapted cells have a more unstable genome and thus the genetic diffusion might be very asymmetric [20, 21]. Our theoretical setting gives us less clear results in the case $d_{1}\neq d_{2}$ because we can’t define and simulate in a simple way a Hamiltonian fitness $r_{H}(x)$ to see were are the optimal traits: the Hamiltonian rather decomposes into

$$ \mathcal {H}(\rho ,N) = \dfrac{d_{1}+d_{2}}{2}\rho ^{2} + r_{H}(x,\rho ) - N,$$

and the function $r_{H}$ then involves the gradient of the solution, which is evolving in space and time.

**Fig. 6:**
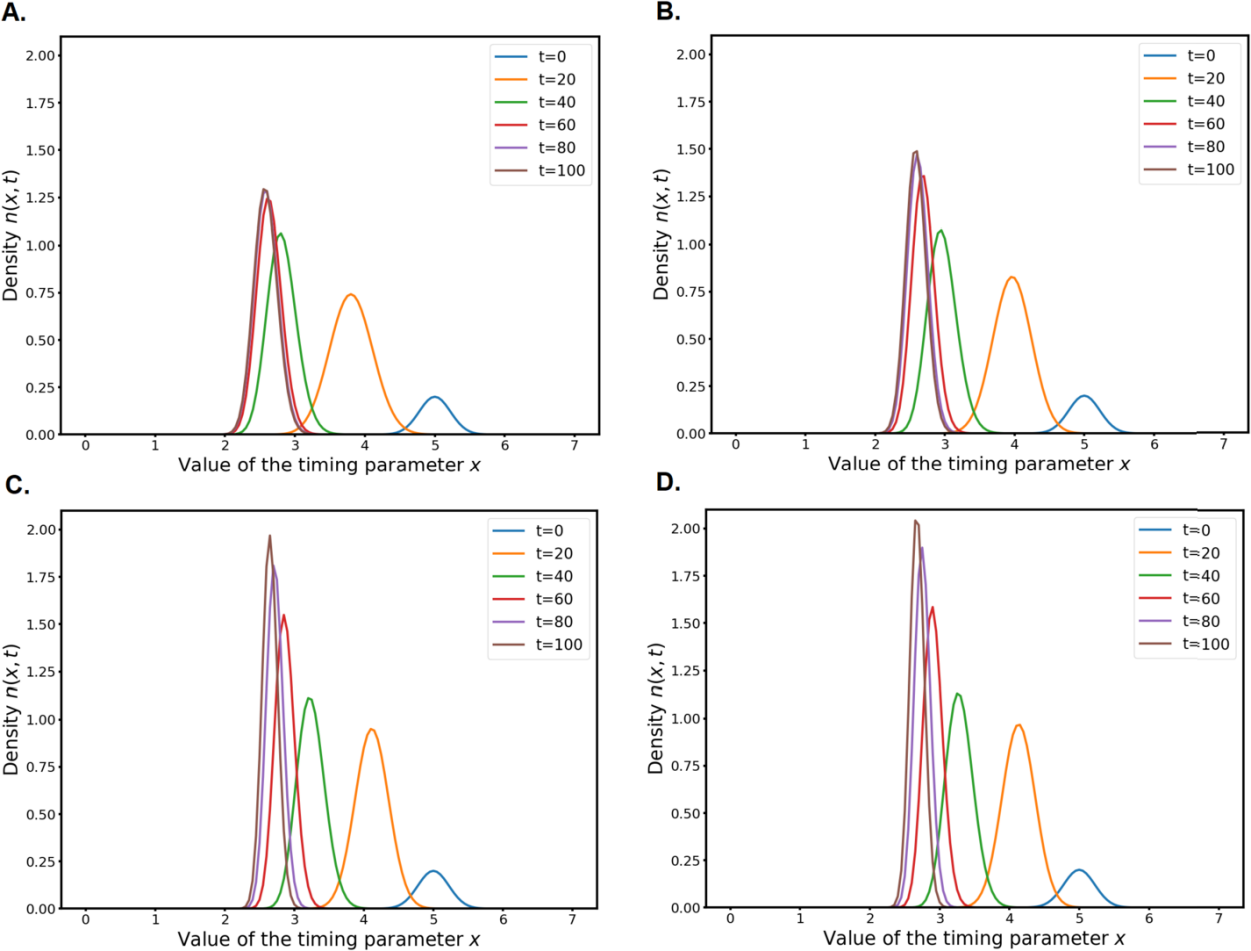
Evolution in time of $n(x,t)$ from the same initial data for $p=3$, $\varepsilon =0.001$ and different pairs $(d_{1},d_{2})$ with same sum $d_{1}+d_{2}$ (A.) $d_{1} = 1$, $d_{2}=1$ (B.) $d_{1} = 0.5$, $d_{2}=1.5$ (C.) $d_{1} = 0.05$, $d_{2}=1.95$ (D.) $d_{1} = 0$, $d_{2}=2$

Therefore, we run numerical experiments for $\varepsilon =0.001$ and different values of $d_{1}$ and $d_{2}$ (see Fig. 6) to see how it impacts the evolution of the solutions in time. It appears that the overall behaviour of the system is not changed much by the different values but asymmetric diffusion make the concentration to a Dirac mass faster. The higher diffusion $d_{2}$ drives the evolution and even in the extreme case $d_{1}=0$ the qualitative behaviour similar to the case $d_{1}=d_{2}=1$. This last case, when the genetic diffusion is assumed to be negligible in healthy cells, is of particular interest for biologists for it allows to investigate adaptation to DNA damage as a mechanism promoting genetic diversity of organisms. Note that this question was opened in [20]. The stability of the model with respect to this particular case strengthens the hypothesis of a role played by adaptation in genetic diversity of cell populations.

### Evolution Along the Parameter $p$ for Fixed $x$

In [44], the authors also explore the influence of the parameter $p$ which describes the slope of the adaptation curve (see Fig. 1B). When $p$ is very large, the population barely adapt before time $x$ since the damage and brutally starts adapting with constant rate after a time $x$. When $p$ is mild, there is a smoother transition and thus cells adapt at more random times. Experimental evidence strongly suggest that the individual adaptation time is heterogeneous and the curves available in the literature suggest that $p$ is neither too small nor too big. In [44], the authors set a biologically realistic value of $x$, and then run the model for different values of $p$ to see which values lead to the fastest recovery of the population after a damage of all the cells. In a stable environment ($\bar{\alpha}(t) = \tfrac{1}{2}$, they find that the optimal value is $p=+\infty $, which is in contrast to experiments. The authors then add randomness into the repair process to account for the fact that repairing can be impossible for some time in hostile environment or if the source of damage is still present. Across different choices of laws for the random environment (Gaussian, exponential, uniform) an optimal value for $p$ emerge. The authors relate this phenomenon to the wider concept of bet-hedging (see [47] for a good introduction to this notion).

In our non-local PDE setting, we add more complexity as now $p$ is not a fixed parameter but a variable and the population is genetically heterogeneous. We simulate the evolution in genetic trait of the population to see if we can validate some of the findings of [44].

#### Stable Environment

**Fig. 7:**
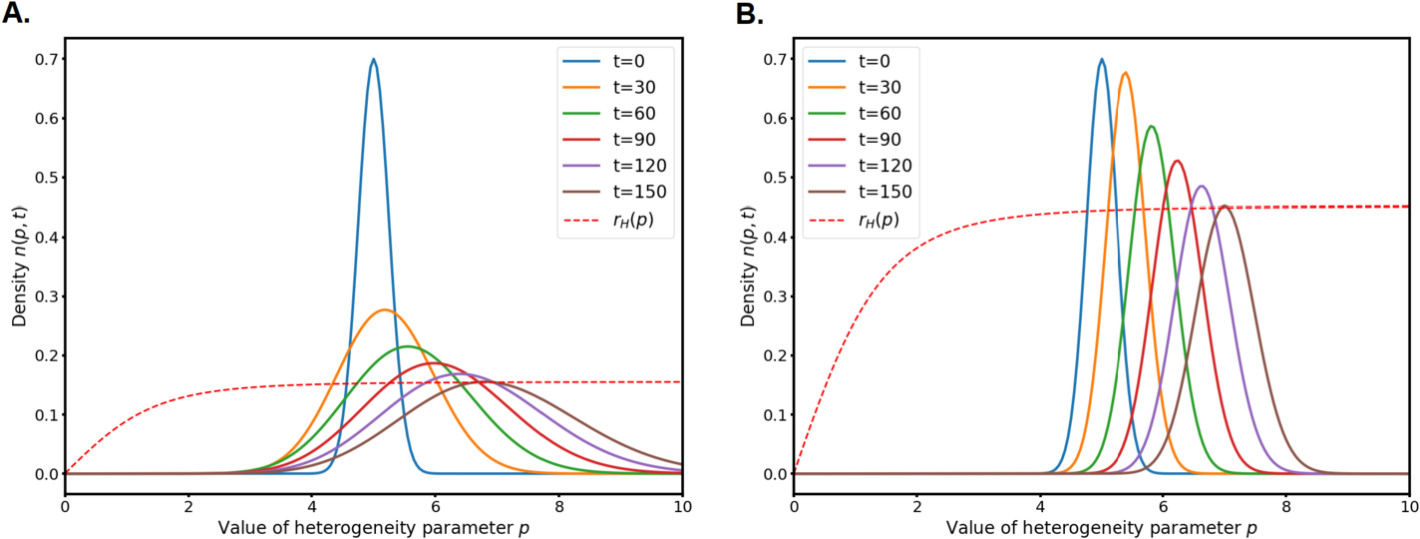
Evolution in time of $n(p,t)$ from the same initial data for $x=4$ and different values of $\varepsilon $. The dashed line is the Hamiltonian fitness $r_{H}(p)$ which is re-scaled for the sake of readability. (A.) $\varepsilon = 0.01$ (B.) $\varepsilon = 0.001$

If we fix a mild value for the timing parameter and take the heterogeneity parameter $p$ as the variable, then the equivalent fitness function $p\mapsto r_{H}(p)$ is increasing. We can observe (see Fig. 7) that the solution stabilizes into a Gaussian moving in time towards $+\infty $. When $\varepsilon $ is smaller, the variance decreases. According to our theoretical results and [5, Theorem 7.1], the solutions concentrate in the limit $\varepsilon \to 0$ on a Dirac measure moving towards infinity. This is what the authors of [5] call the monomorphic case. An increasing fitness function in the Hamilton-Jacobi limit ensures that the movement of the Dirac delta mass is monotone and continuous almost everywhere in time.

This implies that in a stable environment, the cells select an optimal adaptation timing $x^{*}$ for the adaptation to DNA damage and then minimise the variance around it, which amounts to maximising $p$ towards $+\infty $. It confirms the conclusions of [44] obtained with a simpler model: in a predictable environment, the optimal strategy is to minimise randomness and optimise timing.

#### Time-Varying Environment

Yet, the experiments on budding yeast cells show that there is a huge variance around the mean adaptation timing. There are to our knowledge no study quantifying it from a statistical perspective, but the fact that adaptation occur at a variable timing is widely accepted in the community studying it. Following [44], we try to explain this discrepancy between the model and reality by adding a varying environment. In the wild, when sources of damage like heat, solar radiation or chemicals damage the DNA of cells, repair can be impossible for some time because the damage is still present and overloads the repair capacity of the cells. In such case, having some individuals that will adapt earlier can be beneficial for the survival of the population. Since there are no information available about the time distribution of such events in the wild, we choose here to use a time-periodic function for the varying environment, thereby modelling an alternation between favorable and hostile environments. Time periodic environment have been used recently in other studies (see *e.g.* [3, 24]) to model colonisation of an unpredictable environment by organisms.

To make the problem numerically tractable we use

$$ \left \lbrace \begin{aligned} &r_{1}(p,t) = 1 - D + \bar{\alpha}(t)D\int _{0}^{\infty}\alpha (s) e^{- \gamma _{d} s - \int _{0}^{s} \alpha (z)dz - \int _{0}^{s} \beta (p,z)dz}ds , \\ &\delta _{2}(p) = D \int _{0}^{\infty}\beta (s) e^{- \gamma _{d} s - \int _{0}^{s} \alpha (z)dz - \int _{0}^{s} \beta (p,z)dz} d s \end{aligned} \right . $$

rather than

$$ \left \lbrace \begin{aligned} &r_{1}(p,t) = 1 - D + D\int _{0}^{\infty}\alpha (s,t) e^{- \gamma _{d} s - \int _{0}^{s} \alpha (z,t)dz - \int _{0}^{s} \beta (p,z)dz}ds , \\ &\delta _{2}(p,t) = D \int _{0}^{\infty}\beta (s) e^{- \gamma _{d} s - \int _{0}^{s} \alpha (z,t)dz - \int _{0}^{s} \beta (p,z)dz} d s, \end{aligned} \right . $$

because the later requires the program to compute a full vector of integrals at each time step, which makes long time simulations intractable. What this modification change is that the exponential term under the integral in the adaptation and repair inflows are time-independent but the repair inflow is suppressed altogether in hostile environment. It tends to slightly underestimate adaptation when repair is not possible because in the exponential more cells are suppressed “like they have repaired” than the quantity of cells actually repairing. This modification thus leads to a slightly increased death term for damaged cells in hostile environment. It is hard to be sure of which effect it has, but our guess is that the outcome would be the same where we able to simulate the costly time-dependent repair and adaptation rates.

**Fig. 8:**
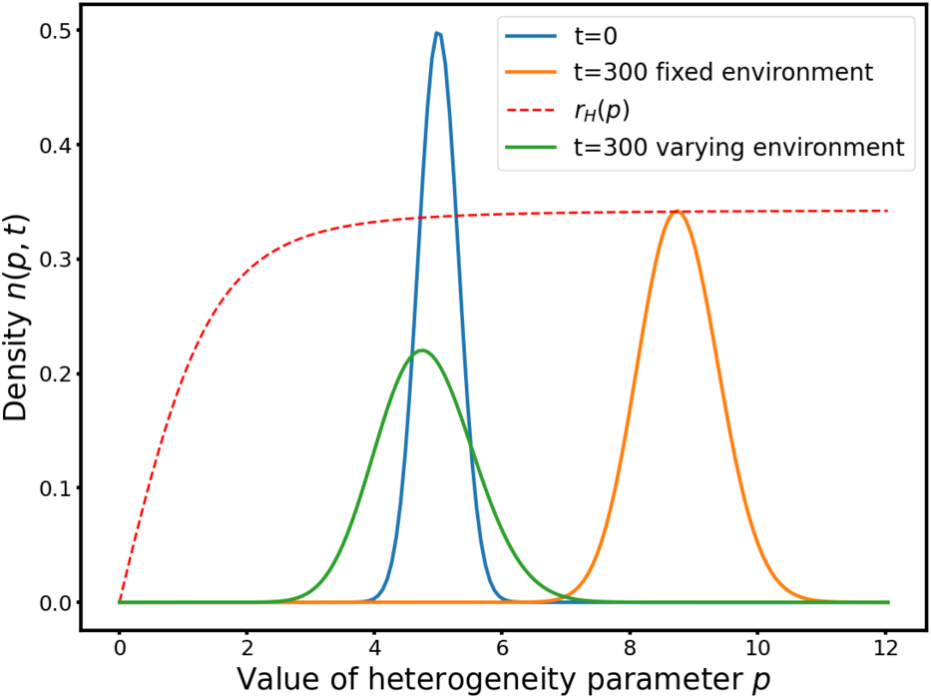
Evolution in time of $n(p,t)$ for $x=4$, $d_{1}=d_{2}=1$ and $\varepsilon =0.001$ in a stable or in a periodically varying environment from the same initial datum

We choose the time-varying environmental function

$$ \bar{\alpha}(t) = \cos \left ( \frac{\pi t}{5} \right )^{8}$$

and we run the model in both a fixed and a time-varying environment from the same initial datum (see Fig. 8). We can observe that at $t=300$ the results are very different. In the case of the stable environment, as in Fig. 7, the mass moves towards $+\infty $. However, with the time-varying environment, the solution moves slowly towards the left. This numerical result strengthens the hypothesis of [44] that the heterogeneity in time of the adaptation to DNA process could be due to a bet-hedging mechanism when cells face an unpredictable environment.

## Conclusion and Perspectives

In this article, we have investigated a cooperative two-population system of non-local parabolic PDEs motivated by a particular application in genetics: the understanding of the so-called *adaptation to DNA damage* phenomenon. We used a Hamilton-Jacobi approach which is well understood for one population non-local models [5, 6, 8]. This approach is well adapted to characterize a guess of the main trait in the limit population as it is underlined in [38]. Such a guess can be useful for instance in the context of cancer treatment as it is developed in [2, 17, 18] (and the references therein). It also appears that studying the Hopf-Cole transform (1.8) $u_{i}$ instead of the solution $n_{i}$ allows to compute the ratio $\frac{n_{i}}{n_{j}}$. It would be helpful to calibrate the system.

First, in order to prove a similar result in our setting, we combined the approach for the one-population model with tools developed in [7] for non-local systems. We wrote the Hamiltonian associated with the system in terms of an eigenvalue of the matrix $\textbf{D}+\textbf{R}$. After performing a Hopf-Cole transform, we first prove uniform regularity results on the solutions $u_{i}^{\varepsilon}$, which allows us to pass to the limit and obtain the constrained Hamilton-Jacobi equation for the limit $u$. When the diffusion coefficients of the two populations are identical, we obtain the additional result that $n_{1}^{\varepsilon}/n_{2}^{\varepsilon}$ converges in time towards a corrective term $q(x)$ dependent only on $r_{1},r_{2},\delta _{1},\delta _{2}$.

Then, we have derived from the ODE model of [44] a PDE system modelling the evolutionary dynamics of adaptation to DNA damage in a population of eukaryotic cells. Our theoretical results and numerical simulations allow us to support the findings of [44] that:

- natural selection could be responsible for the apparition of a precise mean timing for the adaptation phenomenon.
- the experimentally observed heterogeneity of individual adaptation timings in a population of cells could be explained by a bet-hedging mechanism while facing an unpredictable environment.

Our modelling approach is also more general and realistic than the one proposed in [44], because we take into account both mutations and competitions between cells presenting different genotypes in a same environment. It allows us to understand better why a particular trait is selected for the average timing in adaptation to DNA damage: using the optimal average timing allows cells to reproduce and thus out-compete populations with other phenotypes

Yet, this study leaves open several questions on both the mathematical and biological sides that this multiple population approach could help answer in the future.

First, our method relies heavily upon the cross terms $\delta _{1}$ and $\delta _{2}$ being positive. In the case of a competitive system or a prey-predator setting, we cannot apply the same techniques. In particular, it is unlikely that the convergence towards a fixed corrective term $q(x)$ will hold true.

We did not address rigorously the question of the long time behaviour of the system. Our heuristic reasoning and the numerical simulations indicate strongly that there is convergence in time of $n_{1}^{\varepsilon}+n_{2}^{\varepsilon}$ towards a stationary state of the one-population model endowed with the Hamiltonian fitness of the system. A careful analysis is needed to validate this result.

Moreover, the use of a system of non-local PDEs open the possibility to test more hypotheses on adaptation to DNA damage or other natural selection mechanisms. For example by coupling with other equations representing predators, we could sharpen our understanding of the selection dynamics. The presence of dynamically evolving predators densities could introduce an Allee effect, giving ill-adapted cells lesser chances of survival.

Regarding the bet-hedging explanation for the heterogeneity of adaptation to DNA damage, our theoretical framework has to be adapted to prove solid results. It would be very useful to have a theory able to encompass the same kind of system but with time-varying coefficients as in [24] for a single species. The question of periodically changing environment is important in many biological applications [3]. It would be especially important to study theoretically and numerically the influence of the time period of those coefficients with a more systematic and realistic approach. For now, we can conclude with our numerical experiments that the varying environment changes radically the dynamics, but the convergence is very slow. We did experiments for longer time and it was still unclear if a stable equilibrium was reached or not. An inappropriate time-step can also produce strange resonances in the numerical solution, further hindering the numerical exploration. Implementing the asymptotic-preserving scheme recently proposed in [13] could be a route for a better understand of the evolution for small $\varepsilon $ in a variable environment.

It would also be interesting to study this kind of two populations system in higher dimension. In particular, in our biological setting, it would be interesting to have a bi-dimensional space $(x,p)$ for the genetic trait with Neumann boundary conditions on the boundaries $x=0$ and $p=0$ in order to validate the idea that in a stable environment the solution concentrates on a Dirac mass moving at the same time towards the line $(x^{*},p)$ and the direction $p=+\infty $ in the $\epsilon \to 0$ limit. In this bi-dimensional space for the genetic trait, it would also be possible to study the effect of a time-periodic environment in a more realistic framework.

Last, it could be useful to study the more complex model (5.5)–(5.8) theoretically and numerically in order to verify that the quasi-static approximation does not hide key features of the biological phenomenon. This might require cumbersome computations and significant computing power, but it remains feasible in principle with a carefully crafted numerical scheme.

## Appendix A: Existence and Bounds of $N_{\varepsilon}$

We prove in this section the existence of a solution of (1.5). We use the classical Picard Banach fixed point Theorem. The details follow the Appendix A of [8]. We recall that we have made the assumptions (H1)-(H4).

Let $T>0$ be a given time and $\mathcal{A}$ be the following closed subset:

$$ \mathcal{A} := \left \lbrace \begin{pmatrix} n_{1} \\ n_{2} \end{pmatrix} \in C([0,T], (L^{1}_{x}(\mathbb{R}^{+}))^{2}) :\quad n_{i} \geq 0, \quad \int _{\mathbb{R}^{+}} n_{1}(x,t) + n_{2}(x,t) dx \leq a \right \rbrace $$

where $a = \left (\int _{0}^{\infty}n_{1,0}^{\varepsilon}(x) + n_{2,0}^{\varepsilon}(x)dx \right )e^{\frac{C_{R} T}{\varepsilon}}$ and $C_{R} =C_{r} + C_{\delta}$ for some positive $n_{i,0}^{\varepsilon}\in L^{1}(\mathbb{R}^{+})$. In the following, we will denote $\textbf{n}_{0} = \begin{pmatrix} n_{1,0}^{\varepsilon}\\ n_{2,0}^{\varepsilon}\end{pmatrix} $. Next, we define $\Phi $ the following application

$$ \begin{aligned} \Phi \quad : \ & \ \mathcal{A} &&\rightarrow \quad \mathcal{A} \\ &\begin{pmatrix} n_{1} \\ n_{2} \end{pmatrix} &&\mapsto \quad \begin{pmatrix} m_{1} \\ m_{2} \end{pmatrix} \end{aligned} $$

where $\textbf{m } = \begin{pmatrix} m_{1} \\ m_{2} \end{pmatrix} $ is the solution of

A.1 $$ \left \lbrace \begin{aligned} & \partial _{t} \textbf{m} - \varepsilon \textbf{D} \partial _{xx} \textbf{m} = \frac{1}{\varepsilon}\widetilde{\textbf{R}}(x, N_{n}) \textbf{m} \qquad \text{ for } (x,t) \in \mathbb{R}^{+} \times [0,T), \\ &\partial _{x} \textbf{m}(x=0) = 0 \\ &\textbf{m}(t= 0) = \textbf{n}_{0} \end{aligned} \right . $$

where

$$ N_{n} = \int _{0}^{+\infty} (1 \ 1) \textbf{n}(x,t)dx = \int _{0}^{+ \infty}[ n_{1}(x,t) + n_{2}(x,t) ] dx $$

and

$$ \widetilde{\textbf{R}}(x,N) = \left \lbrace \begin{aligned} &\textbf{R}(x, \frac{c_{N}}{2}) && \text{ if } N\leq c_{N}, \\ &\textbf{R}(x,N) &&\text{ if } N \in (\frac{c_{N}}{2}, C_{N}), \\ &\textbf{R}(x, 2C_{N}) && \text{ if } N\geq C_{N}. \end{aligned} \right . $$

The aim is to verify that $\Phi $ satisfies two claims:

1. $\Phi $ maps $\mathcal{A}$ into itself,
2. $\Phi $ is a contractive application for $T$ small enough.

### Proof of claim 1

Let $\textbf{n} \in \mathcal{A}$ and $\textbf{m} = \Phi (\textbf{n})$. By the maximum principle, we have that $m_{i} \geq 0$. It remains to prove the $L^{1}$ bound. According to (A.1), we have

$$ \begin{aligned} \partial _{t} (\int _{0}^{+\infty} m_{1} (x,t) + m_{2}(x,t)dx) &= \frac{1}{\varepsilon } \int _{0}^{+\infty}((r_{1}(x, \widetilde{ N}_{n}) + \delta _{2}(x)) m_{1}(x,t) \\ &\quad{} + (r_{2}(x, \widetilde{ N}_{n}) + \delta _{1}(x)) m_{2}(x,t) ) dx \\ &\leq \frac{C_{R}}{\varepsilon} \int _{0}^{+\infty} m_{1}(x,t) + m_{2}(x,t)dx \end{aligned} $$

with $\widetilde{ N}_{n} = \max (c_{N}, \min (N_{n}, C_{N}))$. Next, we conclude thanks to the Gronwall Lemma that

$$ \begin{aligned} \int _{0}^{+\infty} m_{1} (x,t) + m_{2}(x,t)dx & \leq \left ( \int _{0}^{+ \infty} n_{1,0}^{\varepsilon}(x) + n_{2,0}^{\varepsilon}(x)dx \right ) e^{ \frac{C_{R} t}{\varepsilon}} \\ &\leq \left ( \int _{0}^{+\infty} n_{1,0}^{\varepsilon}(x) + n_{2,0}^{\varepsilon}(x)dx \right ) e^{\frac{C_{R} T}{\varepsilon}} = a. \end{aligned} $$

It finishes the proof of claim 1. □

### Proof of claim 2

Let $\textbf{n}_{1}, \textbf{n}_{2} \in \mathcal{A}$, $\textbf{m}_{1} = \Phi (\textbf{n}_{1})$ and $\textbf{m}_{2} = \Phi (\textbf{n}_{2})$. We have

$$ \partial _{t} (\textbf{m}_{1} - \textbf{m}_{2} ) = \varepsilon \textbf{D} \partial _{xx} (\textbf{m}_{1} - \textbf{m}_{2} )+ \frac{1}{\varepsilon } \widetilde{\textbf{R}}(x, N_{1}) (\textbf{m}_{1} - \textbf{m}_{2}) +\frac{1}{\varepsilon}[\widetilde{\textbf{R}}(x, N_{2}) - \widetilde{\textbf{R}}(x, N_{1}) ] \textbf{m}_{2} .$$

We recall that $\| \textbf{m}_{2} \|_{L^{1}_{x}} \leq a$ and $[\widetilde{\textbf{R}}(x, N_{2}) - \widetilde{\textbf{R}}(x, N_{1}) ] =(N_{1}- N_{2}) \textbf{I}_{2} $. Next, by multiplying at left by $\begin{pmatrix} 1 & 1 \end{pmatrix} $ and integrating over space, we deduce that

$$ \partial _{t} \| \textbf{m}_{1} - \textbf{m}_{2} \|_{L^{1}_{x}} \leq \frac{C_{R}}{\varepsilon} \| \textbf{m}_{1} - \textbf{m}_{2} \|_{L^{1}_{x}} + \frac{a}{\varepsilon} \| \textbf{n}_{1} - \textbf{n}_{2} \|_{L^{1}_{x}}.$$

Since $\| (\textbf{m}_{1} - \textbf{m}_{2})(t=0) \|_{L^{1}_{x}} = 0$, we conclude thanks to the Gronwall Lemma that

$$ \| \textbf{m}_{1} - \textbf{m}_{2} \|_{L^{\infty}_{t}, \ L^{1}_{x}} \leq \frac{\varepsilon a }{C_{R}} (e^{\frac{C_{R} T}{\varepsilon}} - 1) \| \textbf{n}_{1} - \textbf{n}_{2} \|_{L^{\infty}_{t}, \ L^{1}_{x}}. $$

Remarking that $\frac{\varepsilon a }{C_{R}} (e^{\frac{C_{R} T}{\varepsilon}} - 1) \to 0$ as $T \to 0$, we conclude that for $T>0$ small enough, $\Phi $ is contractive. It concludes the proof of existence. □

Next, we focus on the bounds of $N_{\varepsilon}$. According to (1.5), we have

$$ \partial _{t} N_{\varepsilon}(t) =\frac{1}{\varepsilon} \int _{0}^{+ \infty} \begin{pmatrix} 1 & 1 \end{pmatrix} \textbf{R}(x, N_{\varepsilon}) \textbf{n}^{\varepsilon}(x,t)dx.$$

Adding the two first equations of the system (1.5) and integrating over $x$ leads to

$$ \underset{ i, j \in \left \lbrace 1,2 \right \rbrace , \ i \neq j}{\min} ( r_{i}(x) + \delta _{j}(x) -N_{\varepsilon}) \frac{N_{\varepsilon}}{\varepsilon} \leq \partial _{t} N_{\varepsilon}\leq \underset{ i, j \in \left \lbrace 1,2 \right \rbrace , \ i \neq j}{\max} ( r_{i}(x) +\delta _{j}(x) -N_{\varepsilon}) \frac{N_{\varepsilon}}{\varepsilon} .$$

Therefore, since each components of the min (resp. max) in (H3) is decreasing with respect to $N$, we deduce that if $N_{\varepsilon}(t_{0}) = c_{N}$ then $\partial _{t} N_{\varepsilon}(t_{0}) \geq 0$ (resp. if $N_{\varepsilon}(t_{0})= C_{N}$ then $\partial _{t} N_{\varepsilon}(t_{0}) \leq 0$). The conclusion follows.

## Appendix B: Checking the Technical Hypotheses in the Main Application

### Lemma B.1

*Denote*

B.1 $$ A_{\infty}:= \int _{0}^{+\infty} \alpha (s)ds < +\infty $$

*Then*, *there exists* $\kappa \in \mathbb{R}_{+}^{*}$ *such that*

$$ \forall x\in \mathbb{R}_{+}, \ \delta _{2}(x) \geqslant D\mathrm{e}^{-A_{\infty}}(1+e^{-\kappa x})\mathrm{e}^{-2\gamma _{d} x}. $$

### Proof

Note first that the form of $\beta $ implies that

B.2 $$ \exists \kappa \in \mathbb{R}_{+}^{*}, \ \forall x\in \mathbb{R}_{+}^{*}, \ \int _{x}^{2x} \beta (x,s) ds \geqslant \kappa x. $$

Then, we have for all $x\in \mathbb{R}_{+}$,

$$ \delta _{2}(x) \geqslant D\mathrm{e}^{-A_{\infty}}\int _{0}^{+\infty} \beta (x,s)\mathrm{e}^{ - \int _{0}^{s} \beta (x,z)dz}\mathrm{e}^{- \gamma _{d} s} ds. $$

By integration by parts we have

$$\begin{aligned} & \int _{0}^{+\infty}\beta (x,s)\mathrm{e}^{ - \int _{0}^{s} \beta (x,z)dz} \mathrm{e}^{-\gamma _{d} s} ds = 1 - \gamma _{d} \int _{0}^{+\infty} \mathrm{e}^{ - \int _{0}^{s} \beta (x,z)dz}\mathrm{e}^{-\gamma _{d} s} ds \\ &\hspace{3.4cm} = 1 - \gamma _{d}\Big( \underbrace{\int _{0}^{2x} \mathrm{e}^{ - \int _{0}^{s} \beta (x,z)dz}\mathrm{e}^{-\gamma _{d} s} ds}_{:= \omega _{1}(x)} + \underbrace{\int _{2x}^{+\infty}\mathrm{e}^{ - \int _{0}^{s} \beta (x,u)du}\mathrm{e}^{-\gamma _{d} s} ds}_{:= \omega _{2}(x)}\Big). \end{aligned}$$

Moreover,

$$ \omega _{1}(x)\leqslant \int _{2x}^{+\infty}\mathrm{e}^{-\gamma _{d} s} ds = \dfrac{1}{\gamma _{d}}(1-\mathrm{e}^{-2\gamma _{d} x}),$$

and with (B.2),

$$ \omega _{2}(x) \leqslant \int _{2x}^{+\infty}\mathrm{e}^{ - \int _{x}^{2x} \beta (x,z)dz}\mathrm{e}^{-\gamma _{d} s} ds \leqslant \mathrm{e}^{ - \kappa x}\int _{2x}^{+\infty}\mathrm{e}^{-\gamma _{d} s} ds = \dfrac{\mathrm{e}^{-\kappa x}}{\gamma _{d}} \mathrm{e}^{-2\gamma _{d} x} .$$

Thus, we have

$$ \int _{0}^{+\infty}\beta (x,s)\mathrm{e}^{ - \int _{0}^{s} \beta (x,z)dz} \mathrm{e}^{-\gamma _{d} s} ds \geqslant (1-\mathrm{e}^{-\kappa x}) \mathrm{e}^{-2\gamma _{d} x}, $$

and the result follows. □

### Lemma B.2

*Assume* $D \leqslant 1$, *then*

$$ \forall x\in \mathbb{R}_{+}, \ \delta _{2}(x) < 1, $$

*and there exist* $K_{3}, K_{4}\in \mathbb{R}_{+}^{*}$ *such that*

$$ \forall x\in \mathbb{R}_{+}, \ \delta _{2}(x) \leqslant k_{4} \mathrm{e}^{-K_{3} x}. $$

### Proof

Note that there exists two constants $K_{1},K_{2}\in \mathbb{R}_{+}^{*}$ such that

$$ \forall x\in \mathbb{R}_{+}^{*}, \ \int _{0}^{\frac{x}{2}} \beta (x,s) ds \leqslant K_{1} \mathrm{e}^{-K_{2} x}. $$

We have

$$ \delta _{2}(x) \leqslant \int _{0}^{+\infty} \beta (x,s) e^{-\gamma _{d} s - \int _{0}^{s} \alpha (z)dz - \int _{0}^{s} \beta (x,z)dz }ds. $$

By positivity of $\gamma _{d}$ and $A(\cdot )$,

$$ \delta _{2}(x) < \int _{0}^{+\infty} \beta (x,s)\mathrm{e}^{\int _{0}^{s} \beta (x,z)dz }ds = \left [ -\mathrm{e}^{-\int _{0}^{s} \beta (x,z)dz} \right ]_{s=0}^{s=+\infty} = 1, $$

because $\underset{s\to +\infty}{\lim} \int _{0}^{s} \beta (x,z)dz = +\infty $.

We compute:

$$ \delta _{2}(x) < \underbrace{\int _{0}^{\frac{x}{2}} \beta (x,s)\mathrm{e}^{-\gamma _{d} s - \int _{0}^{s} \beta (x,z)dz} ds}_{:= \omega _{1}(x)} + \underbrace{\int _{\frac{x}{2}}^{+\infty} \beta (x,s) \mathrm{e}^{-\gamma _{d} s - \int _{0}^{s} \beta (x,z)dz} ds}_{:= \omega _{2}(x)}. $$

Moreover,

$$ \omega _{1}(x) \leqslant \int _{0}^{\frac{x}{2}} \beta (x,s) ds \leqslant K_{1}\mathrm{e}^{-K_{2} x}, $$

and

$$\begin{array}{c} \omega_{2}(x)⩽e^{-\frac{\gamma_{d}}{2}x}\int_{\frac{x}{2}}^{+\infty }\beta (x,s)e^{\int_{0}^{s}\beta (x,z)dz}ds\\ =e^{-\frac{\gamma_{d}}{2}x}{\left[-e^{-\int_{0}^{s}\beta (x,z)dz}\right]}_{\frac{x}{2}}^{+\infty }=e^{-\frac{\gamma_{d}}{2}x}e^{-\int_{0}^{\frac{x}{2}}\beta (x,s)ds}<e^{-\frac{\gamma_{d}}{2}x}. \end{array}$$

Hence,

$$ \delta _{2}\leqslant (1 + K_{1}) \mathrm{e}^{-\min ( \frac{\gamma _{d}}{2}, K_{2}) x }. $$

 □

## Footnotes

1 The naming “adaptation to DNA damage” can be a bit misleading because it describes a metabolic response of the cells and not a genetic adaptation, so speaking about both adaptation to DNA damage and genetic adaptation from the perspective of evolutionary dynamics can sometimes be confusing. Nonetheless, this expression was used for so long among specialists and has gained so much momentum that it is now impossible to change.

2 Break-induced replication (BIR) consists in healing a one-ended break by copying to the extremity a template chromosome
